# *MLXIPL* associated with tumor-infiltrating CD8+ T cells is involved in poor prostate cancer prognosis

**DOI:** 10.3389/fimmu.2024.1364329

**Published:** 2024-04-18

**Authors:** Yuanming Fan, Yuqiu Ge, Kaiming Niu, Ying Li, Lian-Wen Qi, Haixia Zhu, Gaoxiang Ma

**Affiliations:** ^1^ State Key Laboratory of Natural Medicines, School of Traditional Chinese Pharmacy, China Pharmaceutical University, Nanjing, China; ^2^ Department of Public Health and Preventive Medicine, Wuxi School of Medicine, Jiangnan University, Wuxi, China; ^3^ The Clinical Metabolomics Center, China Pharmaceutical University, Nanjing, China; ^4^ Clinical Laboratory, Tumor Hospital Affiliated to Nantong University, Nantong, China; ^5^ Department of Oncology, Pukou Hospital of Chinese Medicine affiliated to China Pharmaceutical University, Nanjing, China

**Keywords:** prostate cancer, prognosis, CD8+ T cell, MLXIPL, cohort study

## Abstract

**Introduction:**

Within tumor microenvironment, the presence of preexisting antitumor CD8+ T Q7 cells have been shown to be associated with a favorable prognosis in most solid cancers. However, in the case of prostate cancer (PCa), they have been linked to a negative impact on prognosis.

**Methods:**

To gain a deeper understanding of the contribution of infiltrating CD8+ T cells to poor prognosis in PCa, the infiltration levelsof CD8+ T cells were estimated using the TCGA PRAD (The Cancer Genome Atlas Prostate Adenocarcinoma dataset) and MSKCC (Memorial Sloan Kettering Cancer Center) cohorts.

**Results:**

Bioinformatic analyses revealed that CD8+ T cells likely influence PCa prognosis through increased expression of immune checkpoint molecules and enhanced recruitment of regulatory T cells. The MLXIPL was identified as the gene expressed in response to CD8+ T cell infiltration and was found to be associated with PCa prognosis. The prognostic role of MLXIPL was examined in two cohorts: TCGA PRAD (p = 2.3E-02) and the MSKCC cohort (p = 1.6E-02). Subsequently, MLXIPL was confirmed to be associated with an unfavorable prognosis in PCa, as evidenced by an independent cohort study (hazard ratio [HR] = 2.57, 95% CI: 1.42- 4.65, p = 1.76E-03).

**Discussion:**

In summary, the findings suggested that MLXIPL related to tumor-infiltrating CD8+ T cells facilitated a poor prognosis in PCa.

## Introduction

Prostate cancer (PCa) is the most common malignancy in the male urogenital system worldwide (1). As the most common contemporary intervention, radical prostatectomy, radiotherapy, and hormone therapy, have been used for many years. Over the last two decades, the landscape of treatments has changed significantly with the approval of several agents, including chemotherapeutics (docetaxel and cabazitaxel), androgen-receptor signaling inhibitors (abiraterone acetate, enzalutamide, apalutamide, and darolutamide), radioligand therapies (radium-223 and 177Lu-PSMA-617), and PARP-inhibitors (Olaparib). The introduction of them have significantly expanded our therapeutic armamentarium against PCa and contributed to an increased overall survival rate among patients with PCa (37716332) (2, 3). However, advanced PCa, such as metastatic PCa and castration-resistant PCa, continues to pose significant challenges in terms of cure, as suitable therapies are currently lacking (4).

The tumor represents an organ-like structure that emerges from the co-evolution of malignant cells and their immediate environment. The tumor microenvironment (TME) is composed of many different cellular and acellular components. The progression of the tumor, its resistance to therapeutic interventions, as well as invasion and metastasis, are all properties arising from the bidirectional interactions occurring between cancer cells and the TME. Increasing realization of the significance of the TME in cancer biology has shifted cancer research from a cancer-centric model to one that considers the TME as a whole (5, 6). Specially, the TME plays a key role in the procession from primary towards metastatic PCa, in particular bone metastases. Moreover, the interplay between TME and PCa cells is important for AR signaling regulation and response to hormone therapy (7).

Within TME, the presence of preexisting antitumor CD8+ T cells has consistently shown associations with longer disease-free survival and/or overall survival across various cancers with different histological features and anatomical locations. These findings have been observed in both primary tumor settings and metastatic settings (8, 9). Altogether, tumor-infiltrating CD8+ T cells have been consistently associated with a favorable prognosis in the majority of solid cancer types (10). However, in the cases of PCa and clear cell renal cell carcinoma, infiltrating CD8+ T cells have been found to correlate with shorter progression-free survival and overall survival (11, 12). In the case of clear cell renal cell carcinoma, previous studies have confirmed a negative association between the presence of an exhausted phenotype in infiltrating CD8+ T cells and prognosis (13, 14). However, it remains to be established whether a similar pattern exists in PCa.

To comprehend the mechanisms by which infiltrating CD8+ T cells contribute to an unfavorable prognosis in PCa, a comprehensive analysis was conducted. Initially, a comparison was made regarding mutations, immune checkpoint gene expression, and the composition of infiltrating immunoregulatory cells among high and low CD8+ T cells groups. Importantly, potential genes responsive to CD8+ T cells were identified and validated using the independent cohorts. This study may offer novel insights for researchers in understanding the characteristics of CD8+ T cells associated with an unfavorable prognosis in PCa.

## Materials and methods

### Study population

For our independent cohort (NanTong cohort), a total of 94 prostate cancer patients were recruited. All of patients underwent prostate biopsy for diagnosis of prostate cancer. The follow-up protocol involved conducting telephone calls subsequent to the initial diagnosis. Prostate cancer tissues were obtained during tumorectomy procedures and were immediately frozen at -80°C for subsequent analyses. The extraction of tissue RNA was performed in accordance with the manufacturer’s instructions. Clinicopathological findings were assessed based on the tumor–node–metastasis (TNM) classification system. Informed consent was obtained from all patients.

### RNA sequencing and clinical data acquisition

In this study, two publicly available databases were utilized: The Cancer Genome Atlas Prostate Adenocarcinoma (TCGA PRAD) (https://portal.gdc.cancer.gov/) and the Memorial Sloan Kettering Cancer Center (MSKCC) (http://cbio.mskcc.org/cancergenomics/prostate/data/). The TCGA PRAD and MSKCC cohorts were acquired for subsequent analyses. Within the TCGA database, data included transcripts per million (TPM) of RNA sequencing and matched somatic mutation datasets of PCa, were obtained using the TCGA biolinks package in the R software. Additionally, matched tumor purity estimated by immunohistochemistry (IHC) data was downloaded (15), and matched batch information was obtained from https://bioinformatics.mdanderson.org/MQA/. For cases where a gene symbol had multiple expression measurements, the measurement with higher expression was retained. Due to significant RNA degradation in a portion of TCGA PRAD samples, 333 cases were utilized. Ultimately, 282 samples with survival information related to biochemical recurrence were included in our analysis. For the MSKCC cohort sequenced by microarray, normalized log2 mRNA expression data was downloaded.

### Batch effects analysis

Referring to the previous study (16), we selected principal component analysis to analyzed and visualize batch effects of TCGA PRAD.

### Tumor immune microenvironment analysis

The CIBERSORT algorithm, a computational method used to estimate the composition of different immune cell types in a tissue sample from bulk gene expression profiles, was applied to the TCGA PRAD and MSKCC dataset (17). Analyses were performed using 1,000 permutations and default statistical parameters in reference to LM22 matrix. The threshold for categorizing CD8+ T cell infiltration into high and low groups was established by comparing the differences in biochemical recurrence. According to different sequencing methods in this study, the cutoff value was calculated for each data, respectively. The cutoff value selected was the one that yielded the lowest *p*-value. The cytolytic score, which serves as an indicator of local immune cytolytic activity, was calculated as the geometric mean of gene expression values for granzyme A and perforin (18). To estimate T-cell exhaustion level, the murine T-cell exhaustion signature was obtained (19, 20). The murine genes were manually converted to their corresponding human gene equivalents. The degree of T-cell exhaustion was assessed by calculating the mean expression of up-regulated genes minus the mean expression of down-regulated genes.

### Somatic mutation frequency analysis

The somatic mutations were analyzed using the “maftools” R package. The tumor mutation burden (TMB) score was calculated for each patient in accordance with established methods. Oncoplots were utilized to visually present the somatic mutation signatures. The identification of cancer driver genes was achieved through the implementation of the oncodrive CLUST algorithm. Differentially mutated genes were identified by Fisher’s exact test.

### Differentially expressed gene analysis

Before differential expressed gene analysis, the genes with TPM equal to 0 among more than 10% samples were filtered in TCGA PRAD. Differential gene analysis was performed using multiple statistical approaches, including the Wilcoxon rank sum test and signed rank test, DESeq2 and edgeR (21). The genes with an adjusted *p*-value less than 0.05 and an absolute log2 scaled fold change more than 0.5849625 were further analyzed. The false discovery rate (FDR) method was employed for adjusting *p*-values.

### Survival and receiver operating characteristic analyses

The Least Absolute Shrinkage and Selection Operator (LASSO) method was employed to identify stable prognostic candidate genes using biochemical recurrence as the endpoint. The prognostic candidate genes were subsequently validated using the log-rank test, as well as univariate and multivariate Cox proportional hazards regression analysis, with biochemical recurrence as the endpoint. To compute the risk score for each patient, the following formula was utilized:


$$\text{risk score}=\sum_{i=1}^{n}\left[coef\left(mRNAi\right)*Expression\left(mRNAi\right)\right]$$

Risk scores were computed by Cox regression coefficients of the adjusted covariates in both the TCGA and MSKCC cohorts. Additionally, the predictive value of the risk score was evaluated using the area under the receiver operating characteristic curve.

### Functional enrichment analysis

Gene annotation enrichment analyses were conducted on the DEGs between the low and high CD8+ T cell groups using the R package clusterProfiler (22). The analysis included identification of Kyoto Encyclopedia of Genes and Genomes (KEGG) and Gene Ontology (GO) terms. Statistical significance was determined using an adjusted *p*-value cutoff of< 0.05. Additionally, gene set enrichment analysis (GSEA) was performed to identify consistent biological differences between the high and low CD8+ T cell groups, with an adjusted *p*-value cutoff of< 0.05 indicating statistical significance.

### Quantitative real-time polymerase chain reaction

The *MLXIPL* expression in NanTong cohort was measured by qRT-PCR. The relative expression of *MLXIPL* mRNA was calculated by 2^−ΔCt^ method with the normalization to *GAPDH*. Primer sequences of *MLXIPL* were F: AAGATCCGCCTGAACAACG and R: CACTTGTGGTATTCCCGCATC. Primer sequences of *GAPDH* were F: CTGGGCTACACTGAGCACC and R: AAGTGGTCGTTGAGGGCAATG.

### Immunohistochemistry

The immunohistochemistry (IHC) analysis was conducted using rabbit anti-ChREBP (1:200, ab101500, Abcam, Cambridgeshire, England) following the manufacturer’s instructions. Immunostaining intensity was categorized into four grades: 0 (no expression), 1 (mildly positive), 2 (moderately positive), and 3 (markedly positive). The proportion of positive-staining cells was assessed and categorized into five grades: 0 (0%), 1 (1–25%), 2 (26–50%), 3 (51–75%), and 4 (>75%). To generate the IHC score, the percentage of tumor cells showing positivity and the staining intensities were multiplied.

### Statistical analysis

For comparisons between two subtypes, the Wilcoxon rank sum and signed rank tests were employed. Discrete data comparisons were conducted using Fisher’s exact test. Spearman’s correlation analysis was utilized to explore the relationships. All statistical tests were two-sided, and *p*< 0.05 was considered statistically significant unless otherwise stated. The thresholds for *p*-values were set at 0.05, 0.01, and 0.001 (*, ** and ***, respectively). All statistical analyses were performed using R software, version 4.3.1.

## Results

### CD8+ T cell negatively associated with prognosis of PCa

**Figure 1:**
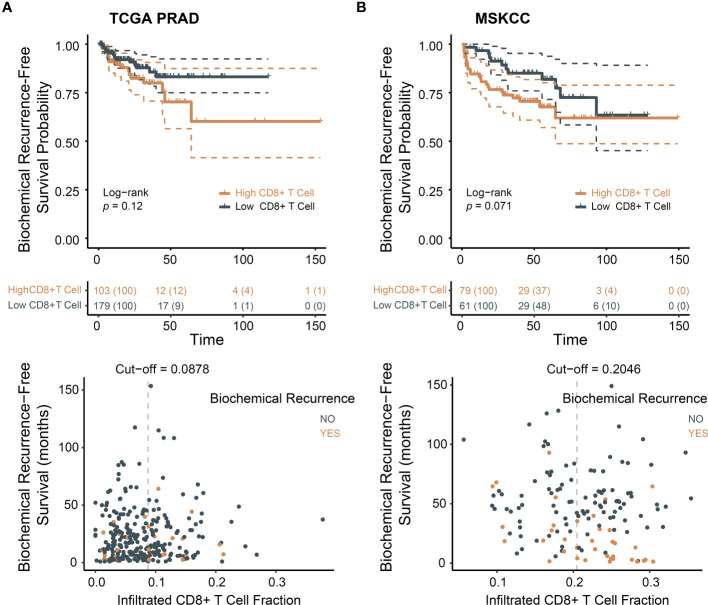
CD8+ T cells infiltration associated with poor prognosis in PCa. **(A)** Biochemical recurrence-free survival for CD8+ T cells in TCGA dataset; **(B)** Biochemical recurrence-free survival for CD8+ T cells in MSKCC dataset.

We investigated the batch effect of TCGA PRAD dataset (**Supplementary Figure S1**). Principal component analysis indicated no batch effects. The baseline characteristics of these two cohorts are present in **Supplementary Table S1**. In line with previous studies (11, 12), patients with higher levels of infiltrated CD8+ T cells exhibited poorer prognosis trend (**Figure 1A**). MSKCC cohort supported this result (**Figure 1B**).

The presence of higher densities of CD8+ T cells has been suggested to be associated with more advanced tumors (10). We examined clinicopathological characteristics, namely age, tumor purity, histopathological subtype, T and N stages, and Gleason score, in relation to the level of CD8+ T cells in TCGA PRAD (**Supplementary Figures S2A–F**) and clinicopathological characteristics, namely age, prostate specific antigen (PSA) level, T and N stages, and Gleason score and *ERG*-fusion status, in relation to the level of CD8+ T cells in MSKCC (**Supplementary Figures S2I–O**). The results indicated that compared with other histopathological type, less CD8+ T cells infiltrated in acinar PCa and age, tumor purity, PSA level, T, N stages, Gleason grade and *ERG*-fusion are not correlated to CD8+ T cell infiltration level.

Given the negative association of exhausted T cells with ccRCC prognosis (13, 14), cytolytic score and T cell exhaustion levels were calculated. Greater immune cytolytic activity was evident in the high CD8+ T cell group (**Supplementary Figures S2G, P**). Comparable levels of exhausted T cells were observed in the TCGA PRAD cohort. In the MSKCC cohort, levels of exhausted T cells were elevated in the high CD8+ T cell group (**Supplementary Figures S2H, Q**). This suggests that T cell exhaustion may partially elucidate the poor prognosis associated with CD8+ T cells.

In summary, our study reaffirmed the observation that CD8+ T cells are linked to an unfavorable prognosis based on available data. Our findings suggest that CD8+ T cells are associated with a poorer prognosis, rather than a higher degree of malignancy resulting in increased infiltration of these T cells.

### CD8+ T cell related poor prognosis is mediated by increased immune check-point genes expression and Tregs

To gain further insights into how CD8+ T cells contribute to the poor prognosis of PCa, we explored whether this involvement is mediated through the TME. Within the TME, immunosuppression can be generated through two possible mechanisms: (1) the presence of differential mutations, which may modulate the immune response in distinct ways; and (2) the presence of tumor-infiltrating T cells that can be suppressed through feedback-induced expression of checkpoint molecules and recruitment of immunoregulatory cells (18).

**Figure 2:**
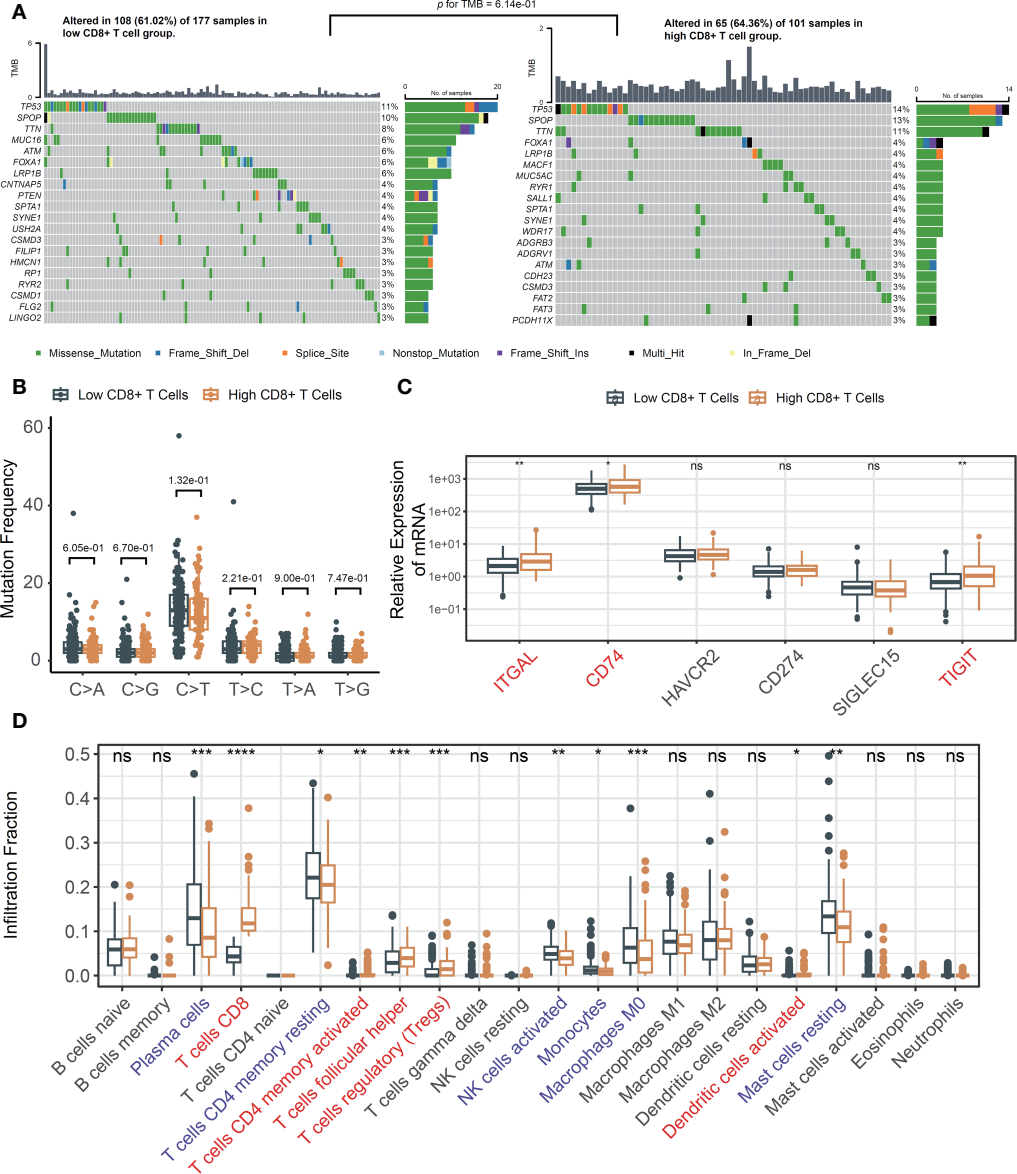
The changes of TME in response to infiltrated CD8+ T cells. **(A)** Oncoplots across tumor-infiltrating CD8+ T cells and comparison of TMBs; **(B)** The mutation frequency analysis of SNPs in TCGA; **(C)** Immune checkpoint genes in low and high infiltrated CD8+ T cell groups; **(D)** The fraction of tumor-infiltrating immune cells across the density of CD8+ T cells. ns, not significant, *p< 0.05, **p< 0.01, ***p< 0.001, ****p< 0.0001.

To investigate the potential role of mutations in mediating the association between CD8+ T cells and poor prognosis in PCa, we conducted somatic mutation frequency analysis. Both the low and high CD8+ T cell groups exhibited low TMB status and similar mutational patterns (**Figure 2A**). High rate of *SPOP*, *TP53*, *TTN* and *FOXA1* mutations were found. Missense mutations were found to be the most prevalent variant classification. Notably, *SPOP* were identified as driver genes despite CD8+ T cell infiltration level (**Supplementary Figure S3A**). SNPs emerged as the most frequent variant type (**Supplementary Figure S3B**). The results showed comparable frequencies of transitions and transversion between the low and high CD8+ T cell groups (**Figure 2B**). Results of Fisher’s exact test confirmed that CD8+ T cell infiltration level was not implicated in gene mutations (**Supplementary Table S2**). Collectively, these results indicate that the association between CD8+ T cells and poor prognosis in PCa is not driven by somatic mutations.

Subsequently, we explored the correlation between immune checkpoint genes and the level of CD8+ T cell infiltration. After filtering the genes utilized in the LM22 matrix, six genes—specifically, *ITGAL*, *CD74*, *HAVCR2*, *CD274*, *SIGLEC15*, and *TIGIT*—were selected based on a review of the literature (23, 24). Increased *ITGAL*, *CD74* and *TIGIT* were observed in the high-density CD8+ T cell subgroup (**Figure 2C**). The relative abundances of 22 infiltrating immune cells were investigated. Notably, elevated immunosuppressive regulatory T cells (Tregs) were observed (**Figure 2D**). These findings suggested that CD8+ T cells may facilitate an unfavorable prognosis through a feedback mechanism involving the abnormality of immune checkpoint gene expression and recruitment or differentiation of immune cells specialized in immune suppression.

### Identification *MLXIPL* mediated by CD8+ T cells

**Figure 3:**
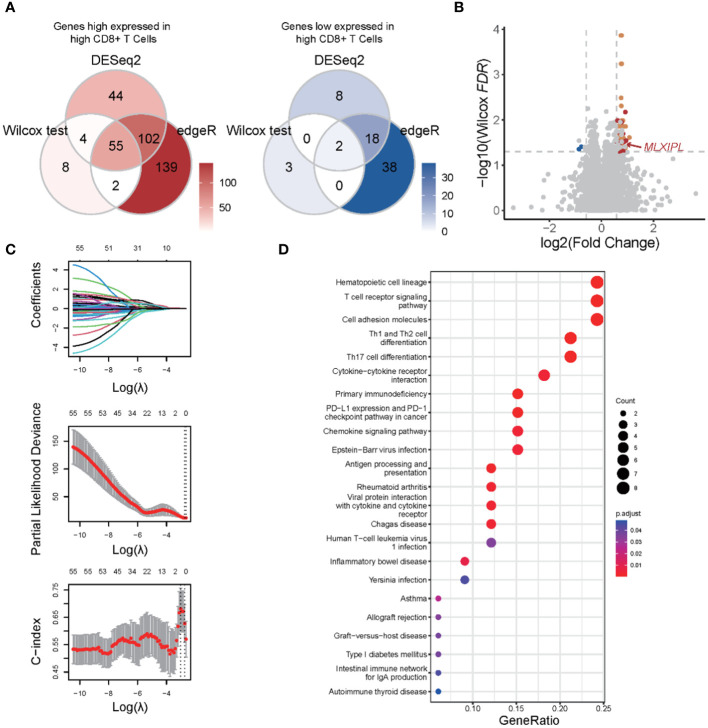
Functional enrichment analysis of differentially expressed genes in response to CD8+ T cells. **(A)** Venn plots of differentially expressed genes identified by Wilcoxon rank sum and signed rank test, DESeq2 and edgeR; **(B)** Volcano plot of differential expression genes; **(C)** LASSO with biochemical recurrence as the endpoint; **(D)** Functional enrichment analysis based on KEGG database.

We hypothesized that protein-coding genes influenced by CD8+ T cells should (1) alter in response to CD8+ T cell infiltration and (2) play a significant role in PCa prognosis. Initially, a differential expression analysis was conducted to compare the low and high CD8+ T cell groups. Three distinct methods, Wilcoxon rank sum test, DESeq2, and edgeR, were utilized. Genes with an adjusted p-value below 0.05 and an absolute log2-scaled fold change exceeding 0.5849625 were identified as DEGs. In total, 74, 233 and 356 DEGs were identified using each respective method. In total, 55 genes exhibited an increase, while 2 genes showed a decrease in the high CD8+ T cell group (**Figures 3A, B**). Among them, 34 genes were involved in the LM22 matrix. Furthermore, we conducted a screening of genes associated with the prognosis of PCa. Among 55 DEGs, *MLXIPL* was identified using LASSO Cox analysis with the TCGA dataset (**Figure 3C**). Specifically, *MLXIPL* showed elevated expression in the high CD8+ T cell group (*p* = 2.06E-4, **Supplementary Figure S4A**) and exhibited a significant correlation with the level of CD8+ T cells (*rho* = 0.25, *p* = 2.92E-5, **Supplementary Figure S4B**). These findings were confirmed using the MSKCC dataset (*p* = 3.81e-10, **Supplementary Figure S4C**; *rho* = 0.67, *p* = 9.54E-20, **Supplementary Figure S4D**).

We conducted a functional enrichment analysis to explore the biological pathways that exhibit alterations in response to CD8+ T cell infiltration. Based on KEGG database, we identified a total of 23 significantly enriched pathways majorly related to such as cell communication, immune responses, and immune checkpoint pathways (**Figure 3D**). The most of significant altered pathways were linked to functions cell communication, antigen presentation, and immune responses. Based on KEGG, GSEA revealed 31 upregulated and 11 downregulated pathways. Consistent with the results of the enrichment analysis, these altered pathways were associated with upregulated cell communication, antigen presentation, and immune responses (**Supplementary Figure S5**). Collectively, these findings suggested that changes in cell communication occur in response to CD8+ T cell infiltration, which may contribute to an unfavorable prognosis of PCa.

### *MLXIPL* mediated by CD8+ T cells facilitated unfavorable prognosis

**Figure 4:**
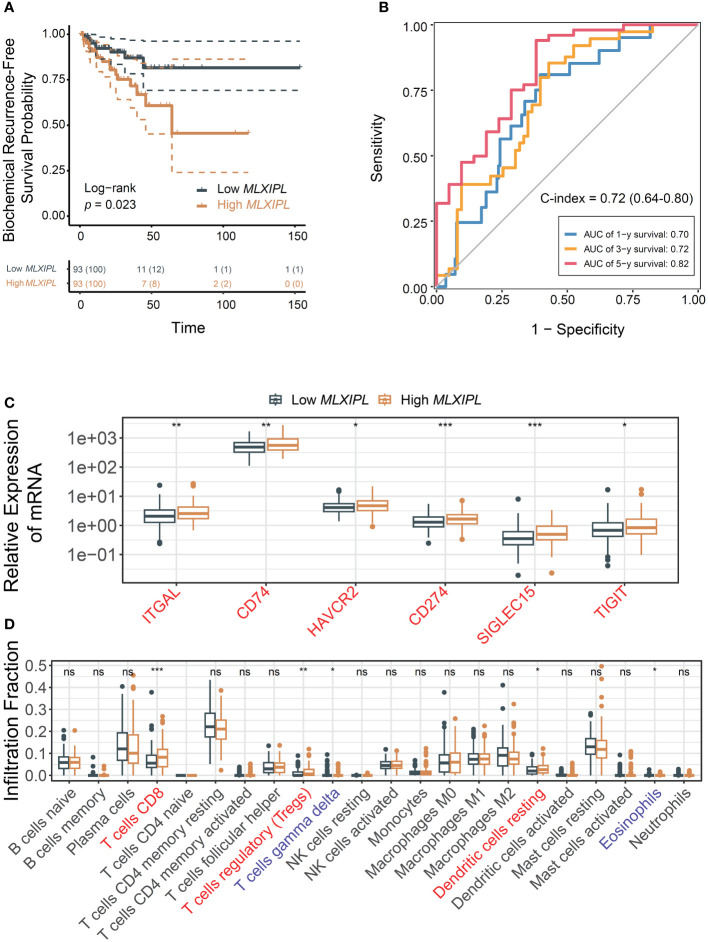
*MLXIPL* induced poor PCa prognosis in TCGA PRAD. **(A)** Survival analysis of biochemical recurrence for *MLXIPL* expression (top tertile *vs* bottom tertile) in TCGA PRAD; **(B)** ROC curves of biochemical recurrence in 1, 3 and 5 year(s); **(C)** Immune checkpoint genes out of LM22 matrix expressed in response to *MLXIPL* expression (top binary *vs* bottom binary); **(D)** The fraction of tumor-infiltrating immune cells across *MLXIPL* expression (top binary *vs* bottom binary). ns, not significant, *p< 0.05, **p< 0.01, ***p< 0.001.

Elevated expression of *MLXIPL* mediated by CD8+ T cells was correlated with an increased rate of biochemical recurrence (log-rank *p* = 2.30E-02, **Figure 4A**). Univariate and multivariate Cox proportional hazards regression models validated that *MLXIPL* significantly contributed to a poor prognosis (crude *p* = 4.20E-03, adjusted *p* = 6.70E-02, **Supplementary Figure S6**). Moreover, the risk score, computed using *MLXIPL*, age, T stage and Gleason score, exhibited strong predictive capabilities for PCa prognosis (1-year AUC = 0.70; 3-year AUC = 0.72; 5-year AUC = 0.82, **Figure 4B**). The C-index value of the model was 0.72 (95% CI: 0.64-0.80).

Consistently, *MLXIPL* remained an independent predictor, unaffected by variables such as age, PSA levels, tumor purity, T and N stages, and Gleason score. In the high *MLXIPL* group, we observed a decreased acinar adenocarcinoma ratio and an increased number of nodes (**Supplementary Figures S7A–F**). Additionally, reduced mutation frequency and TMB were observed in high *MLXIPL* group (**Supplementary Figure S7G**). All TMBs were less than 10 mutations per megabase (MB), indicating a low TMB level. Moreover, somatic mutation frequency analysis revealed no mutation was associated with *MLXIPL* expression (**Supplementary Table S3**). Consistent with the results above, *ITGAL*, *CD74*, and *TIGIT* showed elevated expression in response to *MLXIPL* (**Figure 4C**). Furthermore, *MLXIPL* was associated with the infiltration fraction of several immune cells (**Figure 4D**). Specifically, the high *MLXIPL* group demonstrated an increased infiltration of regulatory T cells.

### Validation the prognostic role of *MLXIPL*

**Figure 5:**
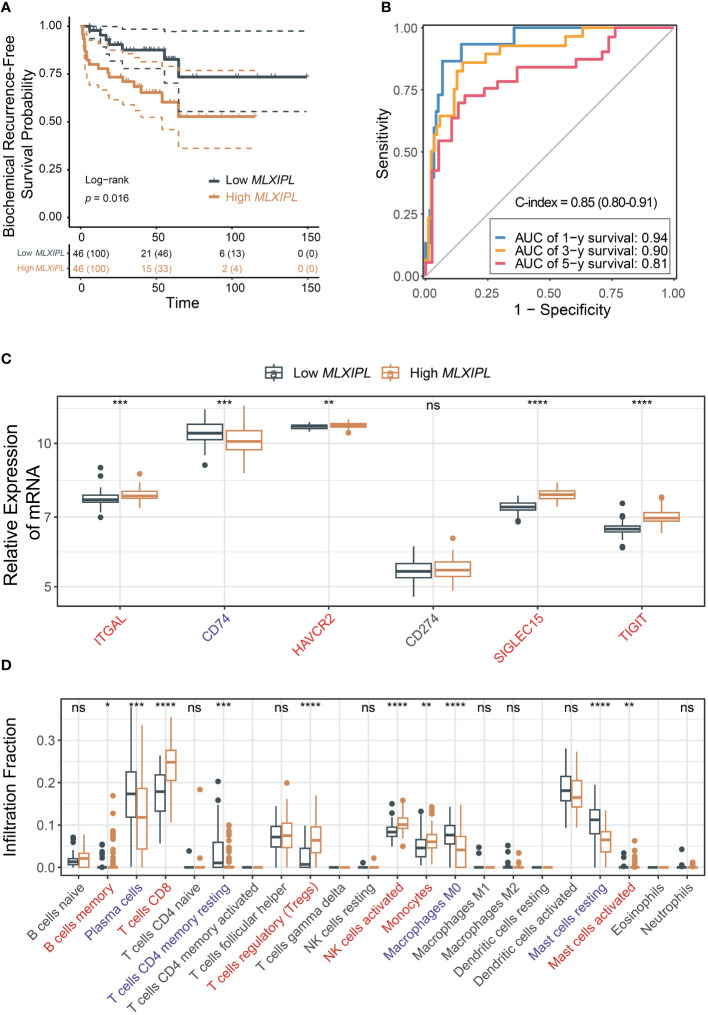
Validation of the role *MLXIPL* in MSKCC cohort. **(A)** Biochemical recurrence-free survival for *MLXIPL* (top tertile *vs* bottom tertile); **(B)** ROC curves of biochemical recurrence in 1, 3 and 5 year(s); **(C)** Immune checkpoint relevant genes out of LM22 matrix expressed in low and high *MLXIPL* groups (top binary *vs* bottom binary); **(D)** The fraction of tumor-infiltrating immune cells across *MLXIPL* expression (top binary *vs* bottom binary). ns, not significant, *p< 0.05, **p< 0.01, ***p< 0.001, ****p< 0.0001.

To validate the findings, we investigated the role of MSKCC in PCa prognosis using the MSKCC cohort. High *MLXIPL* was associated with poor prognosis (log-rank *p* = 1.6E-02, **Figure 5A**). Univariate and multivariate Cox proportional hazards regression models confirmed that *MLXIPL* was associated with poor prognosis (crude *p* = 1.46E-02, adjusted *p* = 4.23E-01, **Supplementary Figure S8**). The AUC values of the risk score, calculated based on *MLXIPL*, T stage, and Gleason score, for predicting 1-, 3-, and 5-year biochemical recurrence were 0.94, 0.90, and 0.81, respectively (**Figure 5B**). The C-index value of the model was 0.85 (95% CI: 0.80-0.91). *MLXIPL* was implicated in N stage, while not correlated to age, PSA level, T stage, Gleason score, metastasis and *ERG*-fusion status (**Supplementary Figure S9**). Four immune checkpoint genes, namely *ITGAL*, *HAVCR2*, *SIGLEC15* and *TIGIT*, increased and *CD74* decreased (**Figure 5C**). Among them, *ITGAL* and *TIGIT* also elevated in TCGA. Additionally, *MLXIPL* were related to infiltration fraction of several immune cells (**Figure 5D**). As anticipated, Tregs infiltration increased in the high *MLXIPL* group.

In summary, these results suggest that CD8+ T cells may modulate *MLXIPL*, thereby affecting PCa prognosis by upregulating *ITGAL* and *TIGIT* and recruiting immunosuppressive Tregs.

### Establishment of the nomogram survival model

Finally, we examined the role of *MLXIPL* in our own cohort, comprising 94 PCa patients with follow-up information (**Supplementary Table S4**). Briefly, the mean ages were 60.02 ± 7.08 and 61.04 ± 7.37 in the low and high *MLXIPL* groups, respectively. The results showed that *MLXIPL* expression was not correlated to age, T stage, N stage and Gleason score (**Supplementary Figure S10**).

**Figure 6:**
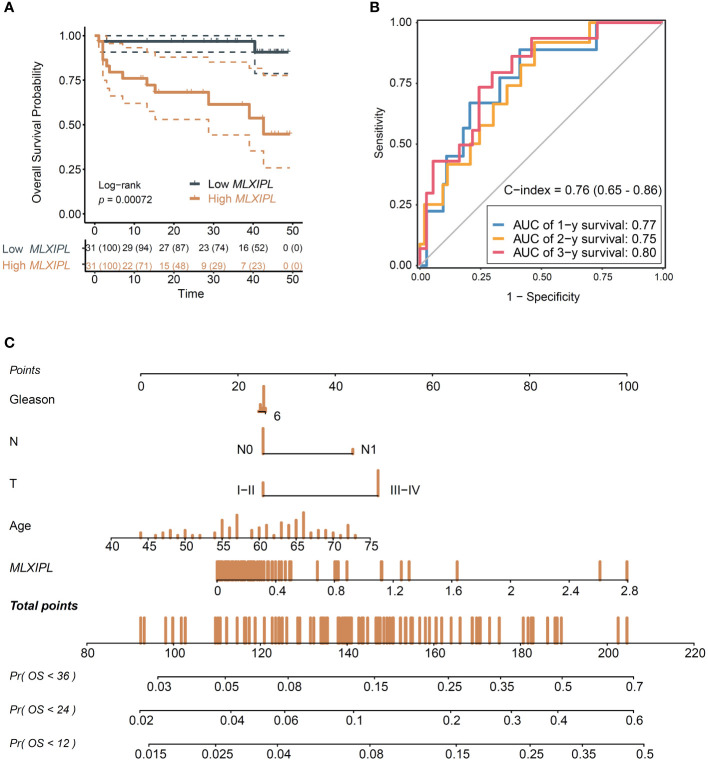
Validation of the role *MLXIPL* in NanTong cohort. **(A)** Overall survival for *MLXIPL* (top tertile *vs* bottom tertile); **(B)** ROC curves of overall survival in 1-, 3- and 5-year(s); **(C)** Nomogram for overall survival of PCa. ROC, receiver operating characteristic.

Consistent with previous findings, *MLXIPL* promoted to poor prognosis (log-rank *p* = 7.2E-04, **Figure 6A**; crude HR = 2.22, 95% CI: 1.33-3.72, *p* = 2.42E-03; adjusted HR = 2.57, 95% CI: 1.42-4.65, *p* = 1.76E-03, **Supplementary Figure S11**). Moreover, when combined with clinicopathologic characteristics, *MLXIPL* demonstrated high predictive performance. The AUC values of the risk score, calculated based on *MLXIPL*, age, T stage, and Gleason score, for predicting 1-, 2-, and 3-year overall survival were 0.77, 0.75, and 0.80, respectively (**Figure 6B**). The C-index value of the model was 0.76 (95% CI: 0.65-0.86). To further improve prognostic prediction, a nomogram model was established using multivariable Cox regression in the NanTong cohort to estimate the 1-, 2-, and 3-year biochemical recurrence, incorporating *MLXIPL* expression, age, T stage, and Gleason grade as variables (**Figure 6C**).

Furthermore, we investigated the protein levels of *MLXIPL* in human prostate tissue microarray to understand its role in PCa tumorigenesis. The baseline characteristics of 59 PCa patients, including 55 pairs of normal adjacent tissue and tumor samples, are presented in **Supplementary Table S5**. In summary, the mean age of the 59 men was 66.83 ± 5.50. IHC results revealed an elevation in MLXIPL protein levels in the tumor tissue (unpaired *p* = 1.03E-2, paired *p* = 3.27E-2, **Supplementary Figure S12**).

## Discussion

This study aimed to comprehend the mechanisms by which infiltrating CD8+ T cells contribute to an unfavorable prognosis in PCa. The findings suggest that the poor prognosis of PCa observed may be attributed to increased expression of immune checkpoint molecules and recruitment of Tregs. Importantly, *MLXIPL* associated with CD8+ T cells was identified and validated. In summary, this study provides new insights into the potential mechanisms by which CD8+ T cells contribute to poor prognosis in PCa.

In this study, we identified *MLXIPL* as a potential downstream target of CD8+ T cells associated with poor prognosis. *MLXIPL* (max-like protein X interacting protein like, also known as Carbohydrate-Responsive Element-Binding Protein, ChREBP) initially identified in 2001, has a key role in regulating metabolic switch (25). The activity of ChREBP is regulated by several mechanisms, including post-translational modifications and interactions with other proteins. For example, phosphorylation of ChREBP by AMP-activated protein kinase (AMPK) under low glucose conditions inhibits its transactivation activity, preventing the induction of lipogenic genes (26). Additionally, ChREBP forms a complex with its partner MLX (Max-like protein X), which is necessary for its DNA-binding activity (27). Understanding these regulatory mechanisms is crucial for elucidating the role of *MLXIPL* in metabolic switches. ChREBP predominantly localizes in the nucleus in response to high glucose levels, which is essential for its function as a transcription factor (28). Under low glucose conditions, ChREBP is primarily found in the cytoplasm, where it remains inactive. This glucose-dependent nuclear-cytoplasmic shuttling is critical for its ability to regulate metabolic pathways adaptively.

Accumulating evidence suggests that *MLXIPL* has a crucial role in cancer pathology and tumorigenesis. Tong et al. observed suppression of *MLXIPL* in hepatoma and colorectal cancer switched aerobic glycolysis to mitochondrial respiration, reduced lipogenesis and nucleotide synthesis and decreased proliferative and tumorigenic potential (28). Through triggering the expression of the PI3K regulatory subunit p85α, *MLXIPL* sustains the activity of the pro-oncogenic PI3K/AKT signaling pathway in hepatocellular cancer. In parallel, increased *MLXIPL* activity reprograms glucose and glutamine metabolic fluxes into fatty acid and nucleic acid synthesis by increasing the expression of genes involved in lipogenesis, glutamine metabolism and *de novo* pyrimidine synthesis to support tumor growth (29). Furthermore, increased MLXIPL staining has been observed in breast cancer, exhibiting a clear positive correlation with malignant progression (30). However, in gastric tumor, *MLXIPL* inhibits proliferation and promotes apoptosis via targeting the cyclin D1-Rb-E2F1 pathway (31). As for PCa, Kaushik et al. reported that *MLXIPL* contribute to CRPC progress in AR-V7-positive 22RV1 cells (32). Given multifaceted role of MLXIPL, the pro-tumorigenic mechanism of *MLXIPL* in PCa may be different from the ones that *MLXIPL* exerts in other tumors. Therefore, the role of *MLXIPL* in PCa remains to be investigated.

The potential mechanisms by which *MLXIPL* is induced in cancer cells by T cell infiltration may be as follows: (1) CD8+ T cells release various cytokines and chemokines upon activation and infiltration into the tumor microenvironment (33). It is possible that one or more of these immune mediators directly or indirectly upregulate *MLXIPL* expression in cancer cells. For example, IFN-γ, a cytokine commonly produced by activated T cells, has been shown to influence the expression of various genes within tumor cells and could potentially modulate *MLXIPL* expression (34). (2) Direct interactions between CD8+ T cells and cancer cells through cell surface receptors and their ligands might play a role in inducing MLXIPL. The engagement of specific immune checkpoints or adhesion molecules could trigger signaling pathways within cancer cells that lead to increased expression of *MLXIPL*. (3) The infiltration of CD8+ T cells and their interaction with other components of the TME, such as fibroblasts, endothelial cells, and other immune cells, could lead to changes in the TME that indirectly promote *MLXIPL* expression in cancer cells. For instance, alterations in hypoxia levels, nutrient availability, or extracellular matrix composition could affect the metabolic state of cancer cells, potentially inducing *MLXIPL* as part of a broader metabolic reprogramming.

To avoid confounding factors, we compared clinicopathological characteristics, TMB, cytolytic scores and exhaustion levels of CD8+ T cells. Most of them were comparative. The density of CD8+ T cells and expression of *MLXIPL* was lower in prostate acinar adenocarcinoma compared to other histopathological subtypes of PCa. Thus, *MLXIPL* may serve as a potential biomarker for the malignant histopathological subtypes of PCa. Moreover, we observed a correlation between *MLXIPL* expression and the number of nodes (N stage) in commonly available data, however, this result cannot be confirmed in the validation cohort. The different results may be account for the difference of race and country. To clarify the role of *MLXIPL* in PCa, further validation across multicenter cohorts is essential.

In the last decade, immunotherapeutic agents have emerged as highly effective therapies for many cancers (35, 36). In patients with advanced PCa, immunotherapy treatments have largely failed (37–39). The disappointing outcomes of immunotherapy treatments in prostate cancer, including immune checkpoint inhibitors and CAR-T cell therapies, can be attributed to several key factors. (1) Low Mutational Burden: PCa typically exhibits a low mutational burden, which may contribute to its poor immunogenicity (40). A low number of neoantigens presented by the tumor cells results in decreased recognition and activation of the immune system against the tumor (41). (2) Immunologically “Cold” Tumor Microenvironment: Prostate cancer often presents an immunologically “cold” microenvironment characterized by limited infiltration and activity of T cells. This environment is less responsive to immunotherapies that rely on the presence and activity of T cells to exert their anti-tumor effects (42). (3) Role of Androgens: Androgens and the AR signaling play a significant role in modulating immune responses. Research indicates that androgens can suppress T cell function and the production of IFNγ, directly affecting the effectiveness of T cell-targeted cancer immunotherapies (43). (4) AR Activity in T Cells: In castration-resistant prostate cancer, AR activity within T cells has been shown to limit the efficacy of checkpoint blockade therapies. Blocking AR signaling can sensitize the tumor-bearing host to effective checkpoint blockade by directly enhancing CD8 T cell function, preventing T cell exhaustion, and improving responsiveness to PD-1 targeted therapy via increased IFNγ expression (43). Given these challenges, strategies combining AR blockade with PD-1/PD-L1 inhibitors have been proposed and shown potential therapeutic effects in some studies (44). Schepisi et al. suggests that the development of CAR-T cell therapies targeting specific prostate cancer antigens could offer a new avenue for treatment (45). These findings underscore the need for a more nuanced understanding of prostate cancer’s unique immune evasion mechanisms and suggest that optimizing treatment may require approaches tailored to these specific challenges. In our study, we postulated that *MLXIPL* expression is associated with the immune responses in PCa. In addition, as a central metabolic coordinator, *MLXIPL* responses to environmental and hormonal signals (25). Thus, inhibiting *MLXIPL* may improve responses of immunotherapy treatments in PCa.

A significant limitation of our study is the absence of direct experimental validation for our findings. While we utilized bioinformatics analyses to explore the role of CD8+ T cells and *MLXIPL* in PCa and validated our findings within our own cohort, experimental validation was not conducted. To replicate the TME, it is essential to use spontaneous tumor models (e.g., *Pten* ^pc–/–^), rather than xenograft or allograft models, or to isolate CD8+ T cells. However, these approaches are time- and cost-intensive. Future research should prioritize incorporating functional assays and mechanistic investigations to strengthen the validity of our results.

## Conclusions

This study unveiled a potential mechanism through which infiltrated CD8+ T cells contribute to a poorer prognosis in PCa. We identified *MLXIPL* as a potential downstream target of CD8+ T cells. *MLXIPL* holds promise as a target to enhance immunotherapy response, and a combination approach involving *MLXIPL* inhibition and immunotherapy may enhance the treatment efficacy for PCa.

## Data availability statement

The original contributions presented in the study are included in the article/**Supplementary Materials**, further inquiries can be directed to the corresponding authors.

## Ethics statement

The studies involving humans were approved by China Pharmaceutical University. The studies were conducted in accordance with the local legislation and institutional requirements. Written informed consent for participation in this study was provided by the participants’ legal guardians/next of kin.

## Author contributions

YF:. YG:. KN: Data curation, Visualization, Writing – review & editing. YL:. LQ:. HZ: Data curation, Visualization, Writing – review & editing. GM: Writing – original draft, Writing – review & editing.
