# Supplementary material for: MLXIPL associated with tumor-infiltrating CD8+ T cells is involved in poor prostate cancer prognosis
Source: Front Immunol. 2024 Apr 18;15:1364329. doi: 10.3389/fimmu.2024.1364329 (PMC11063283; doi:10.3389/fimmu.2024.1364329)

***MLXIPL* Associated with Tumor-infiltrating CD8+ T Cells Is Involved in Poor Prostate Cancer Prognosis**

Yuanming Fan^1†^, Yuqiu Ge^2*^Haixia Zhu^2†^, Ying Li^1^, Lian-Wen Qi^3*^, Gaoxiang Ma^1*^

^1^State Key Laboratory of Natural Medicines, School of Traditional Chinese Pharmacy, China Pharmaceutical University, Nanjing, China.

^2^Clinical Laboratory, Tumor Hospital Affiliated to Nantong University, Nantong, China.

^3^The Clinical Metabolomics Center, China Pharmaceutical University, Nanjing, China.

^4^Department of Public Health and Preventive Medicine, Wuxi School of Medicine, Jiangnan University, Wuxi, China.

*** REPRINTS AND CORRESPONDENCE:** Address reprint requests to Dr. Gaoxiang Ma at the School of Chinese Herbal Medicines of China Pharmaceutical University, E-mail address: [gaoxiang_ma@163.com](mailto:gaoxiang_ma@163.com); Dr. Yuqiu Ge at the Wuxi School of Medicine, Jiangnan University, E-mail address: [yuqiuge0816@163.com](mailto:yuqiuge0816@163.com); Prof. Lian-Wen Qi at the Clinical Metabolomics Center of China Pharmaceutical University, E-mail address: [Qilw@cpu.edu.cn](mailto:Qilw@cpu.edu.cn).

^†^These authors contributed equally to this work

**Running title:** *MLXIPL* and poor prognosis of PCa

**Keywords:** Prostate cancer; CD8+ T Cell; *MLXIPL*; Prognosis

## Supplement Figure 1. Batch effects in TCGA

Batch effects of TCGA PRAD visualized by principal component analysis.

## Supplement Figure 2. Correlation between clinicopathological characteristics and CD8+ T cells

**A)** Age, **B)** Tumor purity, **C)** Tumor subtype, **D)** T stage, **E)** N stage, **F)** Gleason score, **G)** Cytoltic score and **H)** T cell exhaustion score between low CD8+ T and high CD8+ T cell groups in TCGA; **I)** Age, **J)** PSA, **K)** T stage; **L)** N stage, **M)** Gleason score, **N)** Metastasis **O)** *ERG*-fusion status, **P)** cytoltic score and **Q)** T cell exhaustion score between low CD8+ T and high CD8+ T cell groups in MSKCC. CD8+ T Cells were grouped by the cut-off values.

## Supplement Figure 3. Somatic mutation frequency analysis between low and high CD8+ T cell groups

**A)** Identification of cancer genes and **B)** Variant types in low and high CD8+ T cell groups. CD8+ T Cells were grouped by the cut-off values.

## Supplement Figure 4. The correlation of CD8+ T cells and *MLXIPL* expression

**A)** *MLXIPL* expression in high and low CD8+ T cells and **B)** correlation of CD8+ T cells and *MLXIPL* expression in TCGA PRAD; **C)** *MLXIPL* expression in high and low CD8+ T cells and **D)** correlation of CD8+ T cells and *MLXIPL* expression in MSKCC. CD8+ T Cells were grouped by the cut-off values.

## Supplement Figure 5. Altered pathways related to cell communication and immune response mediated by CD8+ T cells based on GSEA

Upregulated antigen processing and presentation, B cell receptor signalling, chemokine signalling, cytokine-cytokine receptor, hematopoietic cell lineage, T cell receptor signalling, Th1 and Th2 cell differentiation and Th17 cell differentiation pathways.

## Supplement Figure 6. Correlation between clinicopathological characteristics and *MLXIPL* expression

**A)** Univariate and **B)** multivariate Cox proportional-hazards regressions for *MLXIPL* and the clinicopathologic characteristics in TCGA PRAD.

## Supplement Figure 7. Clinical indices across *MLXIPL* expression in TCGA cohort

**A)** Age; **B)** Tumor purity; **C)** Histopathological subtype; **D)** T stage; **E)** N stage; **F)** Gleason score and **G)** Mutation frequency analysis with comparison of TMBs between low (bottom binary) and high (up binary) *MLIXPL* groups.

## Supplement Figure 8. Correlation between clinicopathological characteristics and *MLXIPL* expression

**A)** Univariate and **B)** multivariate Cox proportional-hazards regressions for *MLXIPL* and the clinicopathologic characteristics in MSKCC cohort.

## Supplement Figure 9. The role of *MLXIPL* in MSKCC

**A)** Age, **B)** PSA level, **C)** T stage, **D)** N stage, **E)** Gleason score, **F)** Metastasis and **G)** *ERG*-fusion status between low (bottom binary) and high (up binary) *MLXIPL* group.

## Supplement Figure 10. Correlation between clinicopathological characteristics and *MLXIPL* expression

**A)** Age, **B)** T stage, **C)** N stage and **D)** Gleason score between low (bottom binary) and high (up binary) *MLXIPL* groups in NanTong cohort.

## Supplement Figure 11. Correlation between clinicopathological characteristics and *MLXIPL* expression

**A)** Univariate and **B)** multivariate Cox proportional-hazards regressions for *MLXIPL* and the clinicopathologic characteristics in NanTong cohort.

## Supplement Figure 12. Protein levels of *MLXIPL* in normal controls and prostate tumors

**A)** A total of 3 paired representative IHC of normal adjacent prostates (top panels) and prostate tumors (bottom panels); **B)** unpaired and **C)** paired comparisons between normal adjacent tissues and prostate tumors.

## Supplement Table 1. Baseline characteristics of TCGA and MSKCC cohorts

|  | TCGA PRAD  (N = 282) | MSKCC (N = 140) |
| --- | --- | --- |
| **Follow-up time (median, month)** | 17.17 | 45.45 |
| **Biochemical recurrence (N, %)** | 33 （11.70%） | 36 (25.71%) |
| **Age (mean ± sd, year)** | 60.49±6.80 | 57.61±7.00 |
| **Histological type** |  |  |
| Acinar type | 272 | - |
| Other subtype | 10 | - |
| **T** |  |  |
| T2 | 105 | 86 |
| T3 | 169 | 47 |
| T4 | 4 | 7 |
| **N** |  |  |
| N0 | 205 | 105 |
| N1 | 43 | 12 |
| **Gleason Score** |  |  |
| 6 | 21 | 42 |
| 7 | 152 | 77 |
| 8 | 38 | 11 |
| 9 | 69 | 10 |
| 10 | 2 | 0 |
| **Metastasis (N, %)** | 0 (0.00%) | 9 (6.43%) |
| ***ERG*-fusion status** |  |  |
| Negative | - | 86 |
| Flat | - | 22 |
| Positive | - | 32 |
| **Race** |  |  |
| White | 232 | 106 |
| Black | 35 | 28 |
| Asian | 7 | 2 |

## Supplement Table 2. Differentially mutated genes across tumor-infiltrating CD8+ T cells in TCGA

| **Hugo Symbol** | **Low CD8+ T Cells** | **High CD8+ T Cells** | ***p*-value** | **OR** | **Up CI** | **Low CI** | **adjusted *p*-value** |
| --- | --- | --- | --- | --- | --- | --- | --- |
| *FILIP1* | 6 | 0 | 8.97E-02 | Inf | Inf | 0.68 | 6.83E-01 |
| *MUC16* | 11 | 2 | 1.43E-01 | 3.27 | 30.95 | 0.69 | 6.83E-01 |
| *CSMD1* | 5 | 0 | 1.63E-01 | Inf | Inf | 0.53 | 6.83E-01 |
| *FLG2* | 5 | 0 | 1.63E-01 | Inf | Inf | 0.53 | 6.83E-01 |
| *LINGO2* | 5 | 0 | 1.63E-01 | Inf | Inf | 0.53 | 6.83E-01 |
| *CNTNAP5* | 7 | 1 | 2.65E-01 | 4.10 | 187.25 | 0.51 | 7.96E-01 |
| *PTEN* | 7 | 1 | 2.65E-01 | 4.10 | 187.25 | 0.51 | 7.96E-01 |
| *ATM* | 10 | 3 | 3.87E-01 | 1.95 | 11.30 | 0.49 | 9.21E-01 |
| *HMCN1* | 6 | 1 | 4.28E-01 | 3.50 | 162.87 | 0.42 | 9.21E-01 |
| *USH2A* | 7 | 2 | 4.95E-01 | 2.03 | 20.44 | 0.38 | 9.21E-01 |
| *TTN* | 15 | 11 | 5.26E-01 | 0.76 | 1.91 | 0.31 | 9.21E-01 |
| *SPOP* | 18 | 13 | 5.54E-01 | 0.77 | 1.79 | 0.34 | 9.21E-01 |
| *TP53* | 20 | 14 | 5.70E-01 | 0.79 | 1.79 | 0.36 | 9.21E-01 |
| *RP1* | 6 | 2 | 7.15E-01 | 1.73 | 17.89 | 0.30 | 1 |
| *FOXA1* | 10 | 4 | 7.76E-01 | 1.45 | 6.51 | 0.40 | 1 |
| *LRP1B* | 10 | 4 | 7.76E-01 | 1.45 | 6.51 | 0.40 | 1 |
| *CSMD3* | 6 | 3 | 1 | 1.15 | 7.24 | 0.24 | 1 |
| *MUC17* | 5 | 3 | 1 | 0.95 | 6.25 | 0.18 | 1 |
| *RYR2* | 6 | 3 | 1 | 1.15 | 7.24 | 0.24 | 1 |
| *SPTA1* | 7 | 4 | 1 | 1.00 | 4.77 | 0.25 | 1 |
| *SYNE1* | 7 | 4 | 1 | 1.00 | 4.77 | 0.25 | 1 |

CD8+ T Cells were grouped by the cut-off values. CI, confidence interval; OR, odds ratio.

## Supplement Table 3. Differentially mutated genes across *MLXIPL* expression in TCGA

| **Hugo Symbol** | **Low *MLXIPL*** | **High *MLXIPL*** | ***p*-value** | **OR** | **Up CI** | **Low CI** | **adjusted *p*-value** |
| --- | --- | --- | --- | --- | --- | --- | --- |
| *KDM6A* | 7 | 0 | 1.45E-02 | Inf | Inf | 1.46 | 2.90E-01 |
| *ATM* | 10 | 3 | 8.48E-02 | 3.45 | 19.93 | 0.86 | 5.75E-01 |
| *SPOP* | 20 | 11 | 1.27E-01 | 1.92 | 4.63 | 0.84 | 5.75E-01 |
| *TTN* | 18 | 10 | 1.62E-01 | 1.88 | 4.76 | 0.79 | 5.75E-01 |
| *CNTNAP5* | 7 | 2 | 1.73E-01 | 3.56 | 35.78 | 0.66 | 5.75E-01 |
| *RP1* | 7 | 2 | 1.73E-01 | 3.56 | 35.78 | 0.66 | 5.75E-01 |
| *SPTA1* | 8 | 3 | 2.17E-01 | 2.72 | 16.25 | 0.63 | 6.21E-01 |
| *PTEN* | 6 | 2 | 2.82E-01 | 3.03 | 31.25 | 0.53 | 6.70E-01 |
| *MUC17* | 3 | 6 | 3.33E-01 | 0.48 | 2.32 | 0.08 | 6.70E-01 |
| *CSMD3* | 7 | 3 | 3.35E-01 | 2.36 | 14.45 | 0.53 | 6.70E-01 |
| *HMCN1* | 5 | 2 | 4.47E-01 | 2.51 | 26.80 | 0.40 | 8.13E-01 |
| *TP53* | 16 | 18 | 7.17E-01 | 0.86 | 1.88 | 0.39 | 9.24E-01 |
| *KMT2C* | 4 | 5 | 7.48E-01 | 0.78 | 3.73 | 0.15 | 9.24E-01 |
| *RYR2* | 4 | 5 | 7.48E-01 | 0.78 | 3.73 | 0.15 | 9.24E-01 |
| *USH2A* | 4 | 5 | 7.48E-01 | 0.78 | 3.73 | 0.15 | 9.24E-01 |
| *SYNE1* | 5 | 6 | 7.69E-01 | 0.82 | 3.29 | 0.19 | 9.24E-01 |
| *LRP1B* | 8 | 6 | 7.85E-01 | 1.33 | 4.79 | 0.39 | 9.24E-01 |
| *MUC16* | 7 | 6 | 1 | 1.16 | 4.29 | 0.32 | 1 |
| *FOXA1* | 7 | 7 | 1 | 0.99 | 3.39 | 0.29 | 1 |
| *KMT2D* | 5 | 5 | 1 | 0.99 | 4.39 | 0.22 | 1 |
| *KDM6A* | 7 | 0 | 1.45E-02 | Inf | Inf | 1.46 | 2.90E-01 |

*MLXIPL* expression was grouped by the median. CI, confidence interval; OR, odds ratio.

## Supplement Table 4. Baseline characteristics of NT cohort

|  | ***MLXIL* low**  **(N = 47)** | ***MLXIPL* high**  **(N = 47)** | ***p*-value** |  |
| --- | --- | --- | --- | --- |
| **Follow-up time (median, month)** | 36.00 | 20.50 |  |  |
| **Biochemical recurrence (N, %)** | 4 (8.51%) | 13 (27.66%) | **3.21E-02** | † |
| **Age (mean ± sd, year)** | 60.02±7.08 | 61.04±7.37 | 4.95E-01 | * |
| **T stage (N, %)** |  |  | 2.76E-01 | † |
| I-II | 19 (40.43%) | 13 (27.66%) |  |  |
| III-IV | 28 (59.57%) | 34 (72.34%) |  |  |
| **N stage (N, %)** |  |  | 7.72E-01 | † |
| N0 | 39 (41.49%) | 41 (43.62%) |  |  |
| N1 | 8 (8.51%) | 6 (6.38%) |  |  |
| **Gleason Score (N, %)** |  |  | 8.00E-02 | † |
| < 7 | 2 (4.26%) | 6 (12.77%) |  |  |
| = 7 | 35 (74.47%) | 25 (53.19%) |  |  |
| > 7 | 10 (21.28%) | 16 (34.04%) |  |  |
| **Residual tumor** |  |  | 5.45E-01 | † |
| R0 | 37 (39.36%) | 36 (38.30%) |  |  |
| R1 | 9 (9.57%) | 11 (11.70%) |  |  |
| RX | 1 (1.06%) | 0 (0.00%) |  |  |
| **Radiotherapy** |  |  | 5.45E-01 | † |
| NO | 37 (39.36%) | 29 (30.85%) |  |  |
| YES | 3 (3.19%) | 5 (5.32%) |  |  |
| **Chemotherapy** |  |  | 1.00E+00 | † |
| NO | 37 (39.36%) | 31 (32.98%) |  |  |
| YES | 3 (3.19%) | 3 (3.19%) |  |  |

*MLXIPL* expression was grouped by the median. Values are mean±SD or %; * *p* values were calculated by Student’s *t* test for continuous characteristics; † *p* values were calculated by Chi-square test for binary characteristics.

## Supplement Table 5. Baseline characteristics of Human Prostate Tissue Microarray

|  | **PCa patients (N = 58)** |
| --- | --- |
| **Age (mean ± sd, year)** | 66.83±5.50 |
| **Gleason Grade** |  |
| < 7 | 18 |
| = 7 | 35 |
| > 7 | 1 |

**Supplement Figure 1**


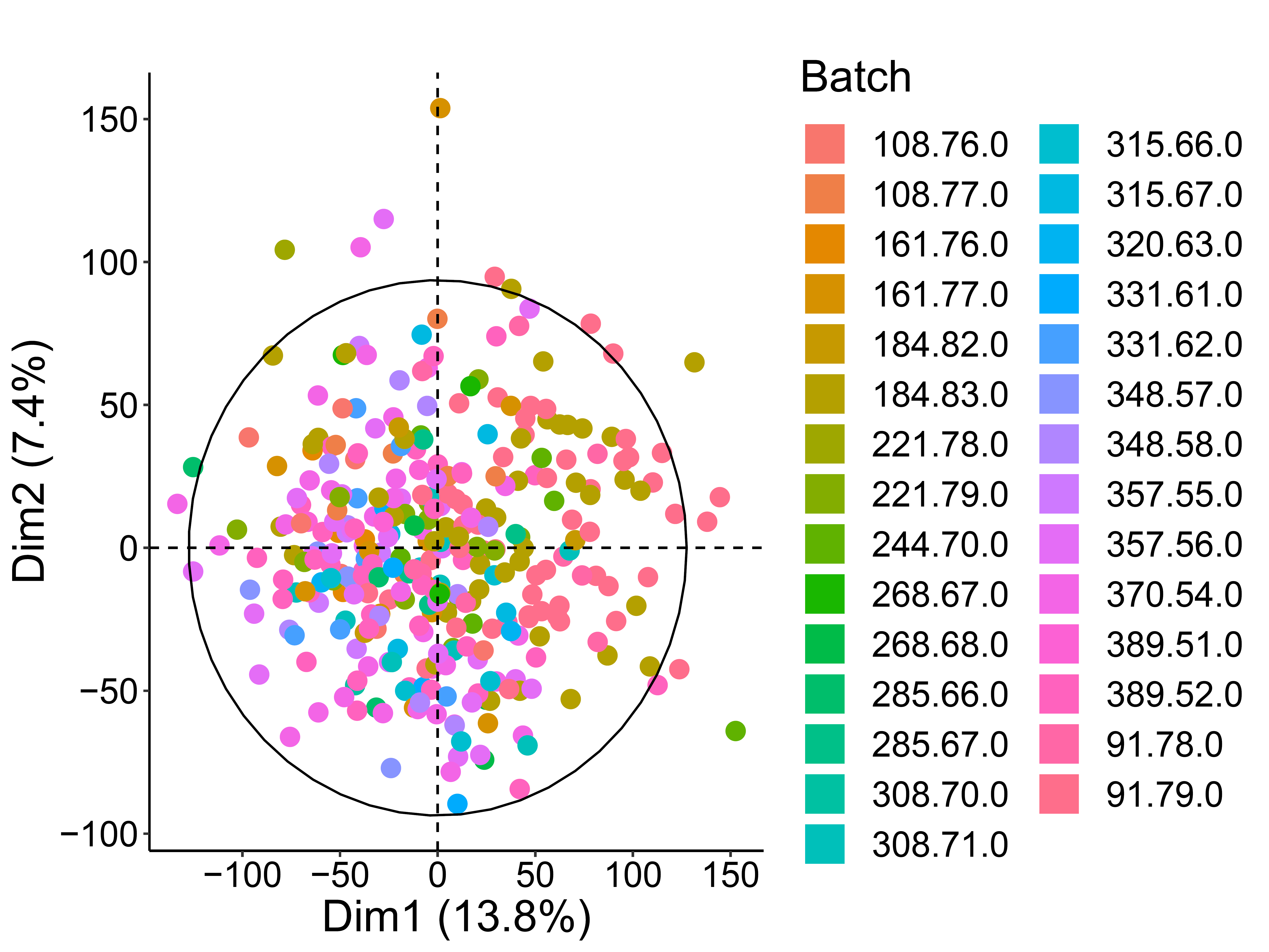


**Supplement Figure 2**


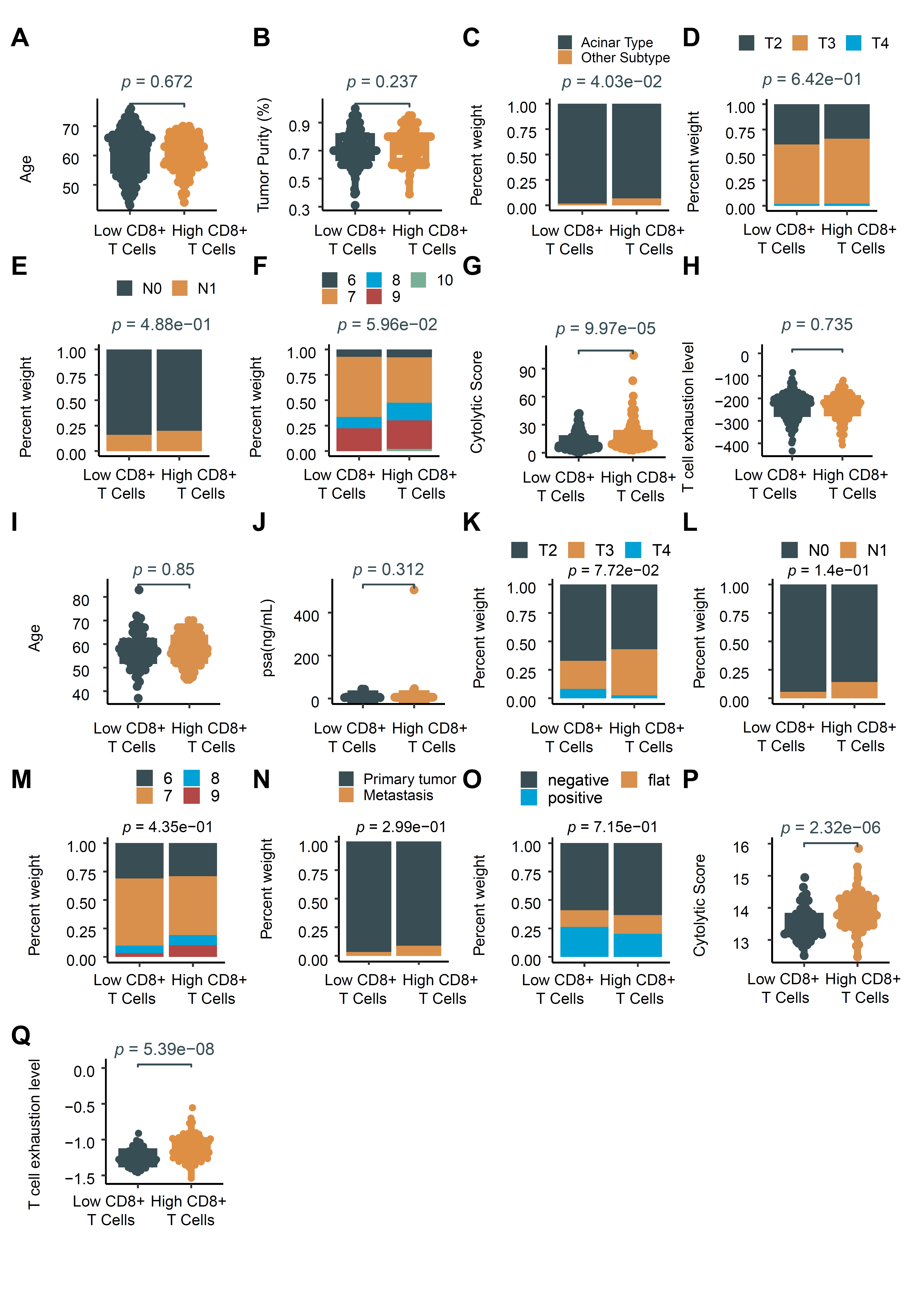


**Supplement Figure 3**


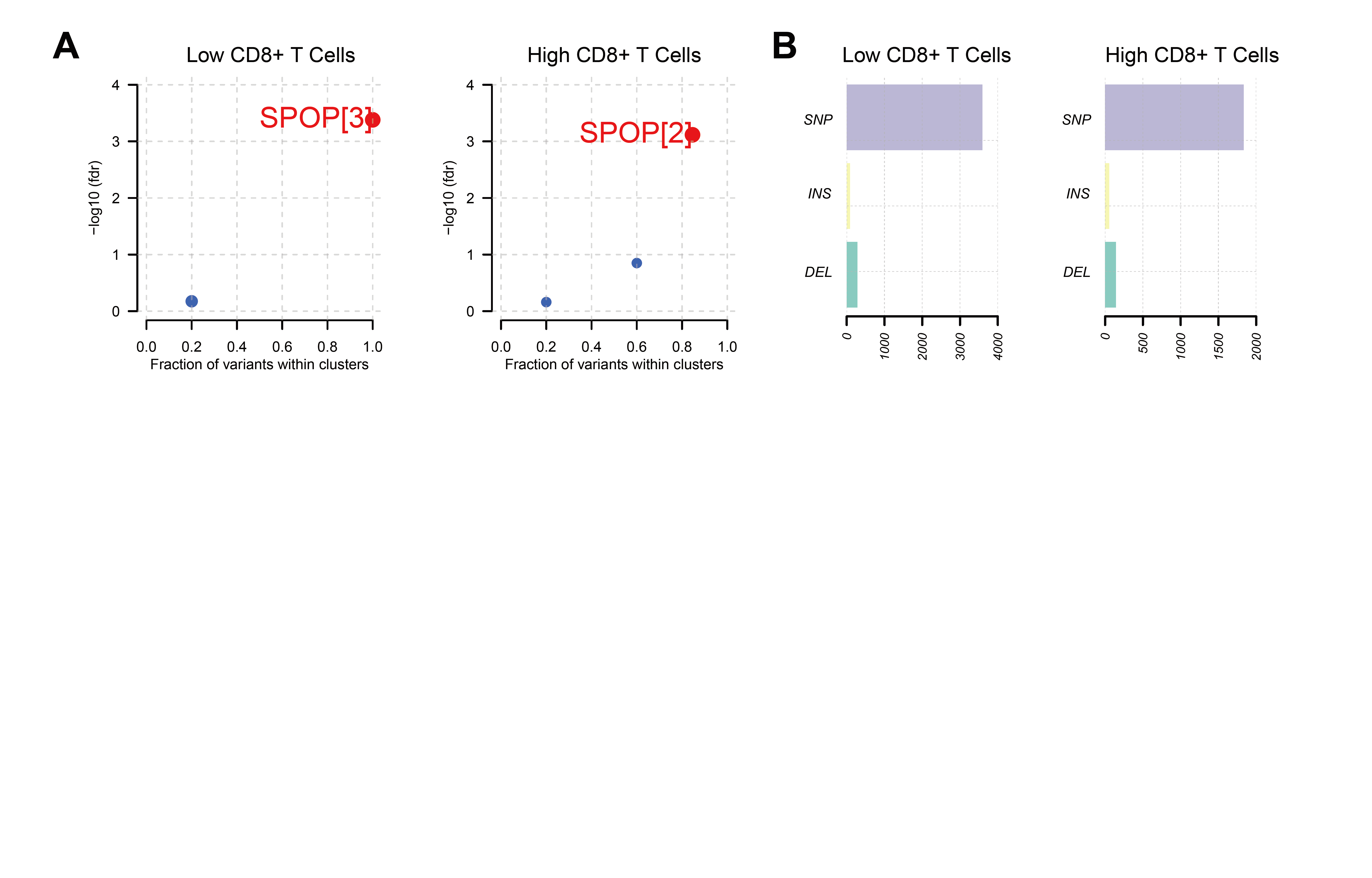


**Supplement Figure 4**


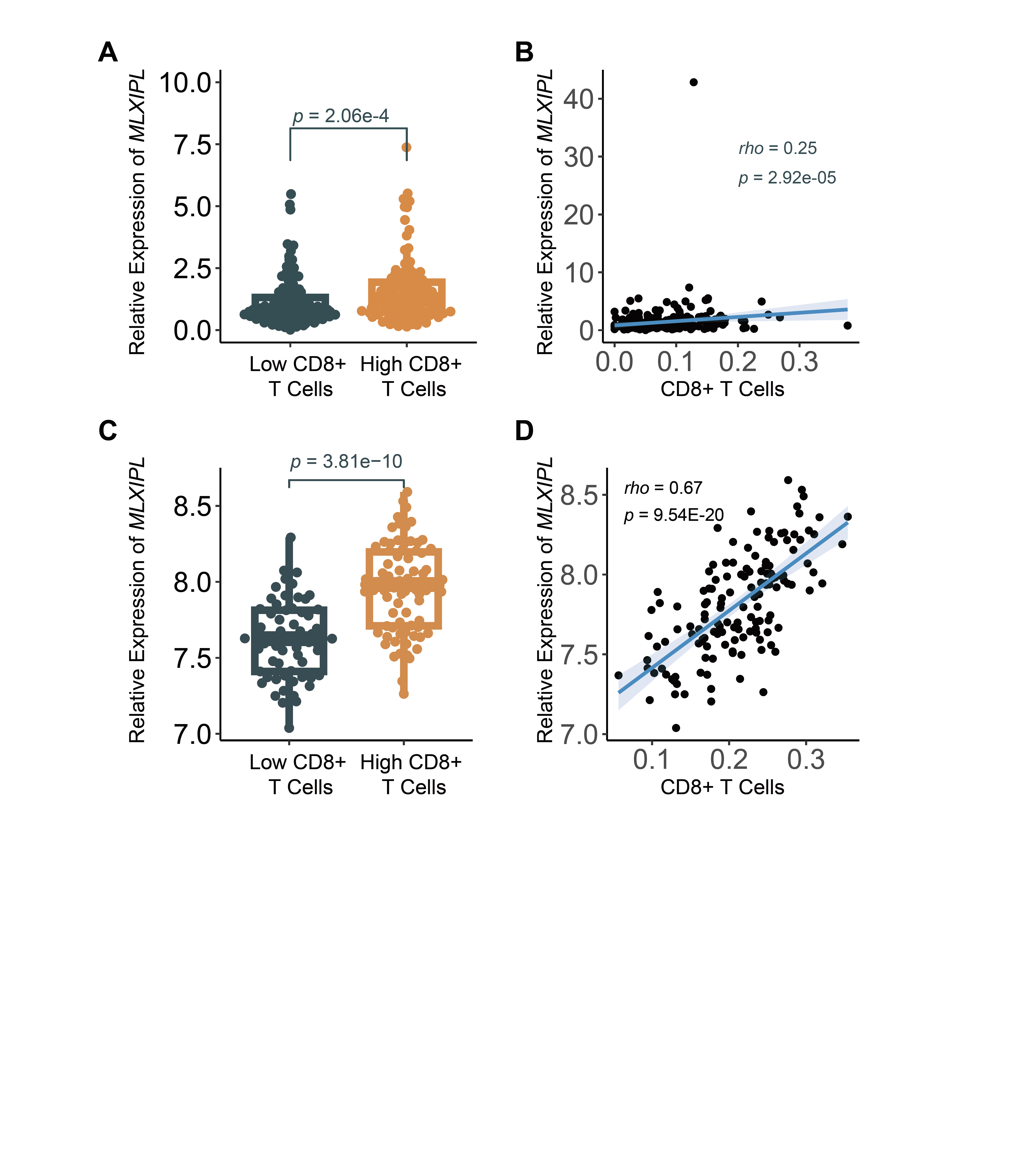


**Supplement Figure 5**


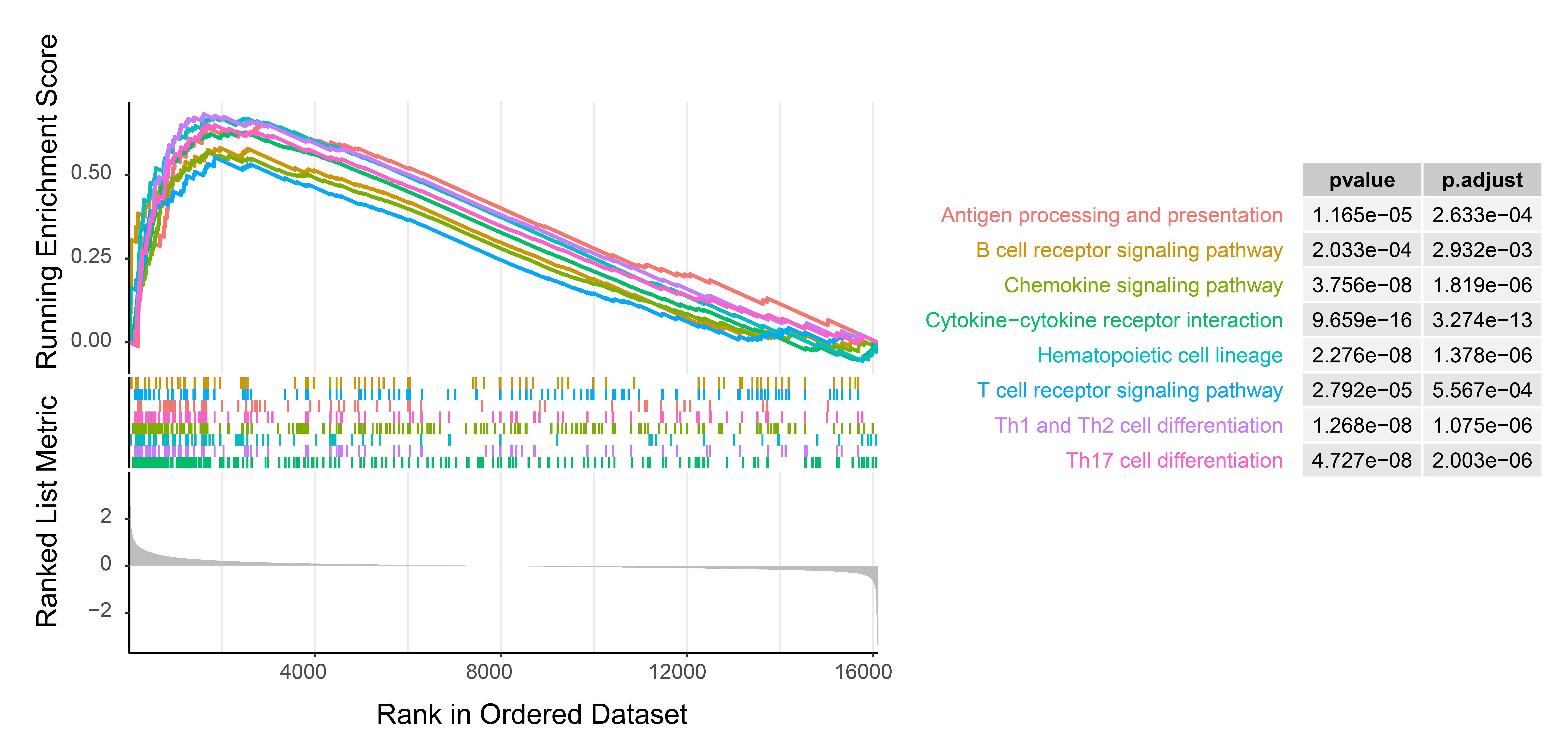


**Supplement Figure 6**


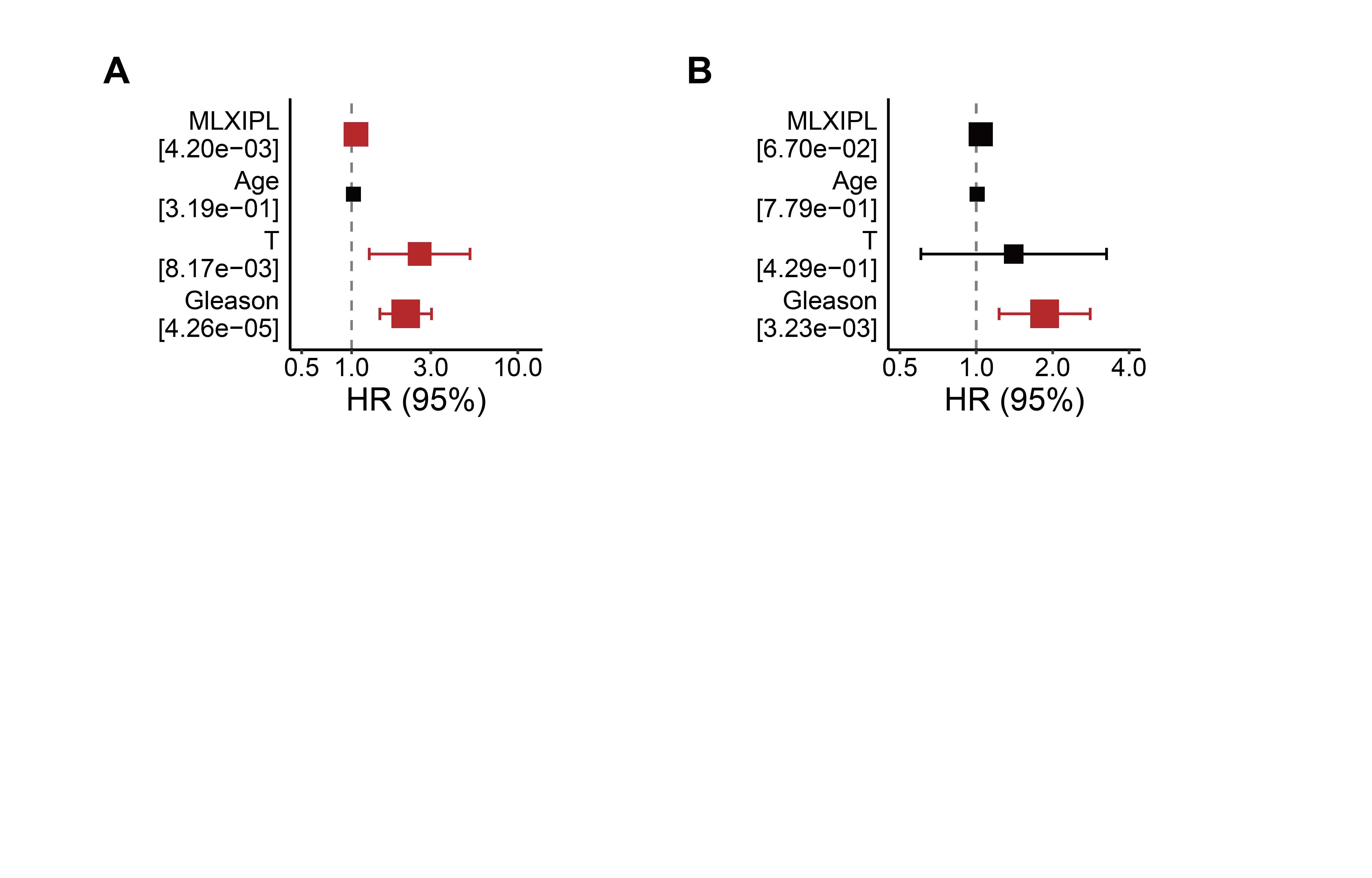


**Supplement Figure 7**


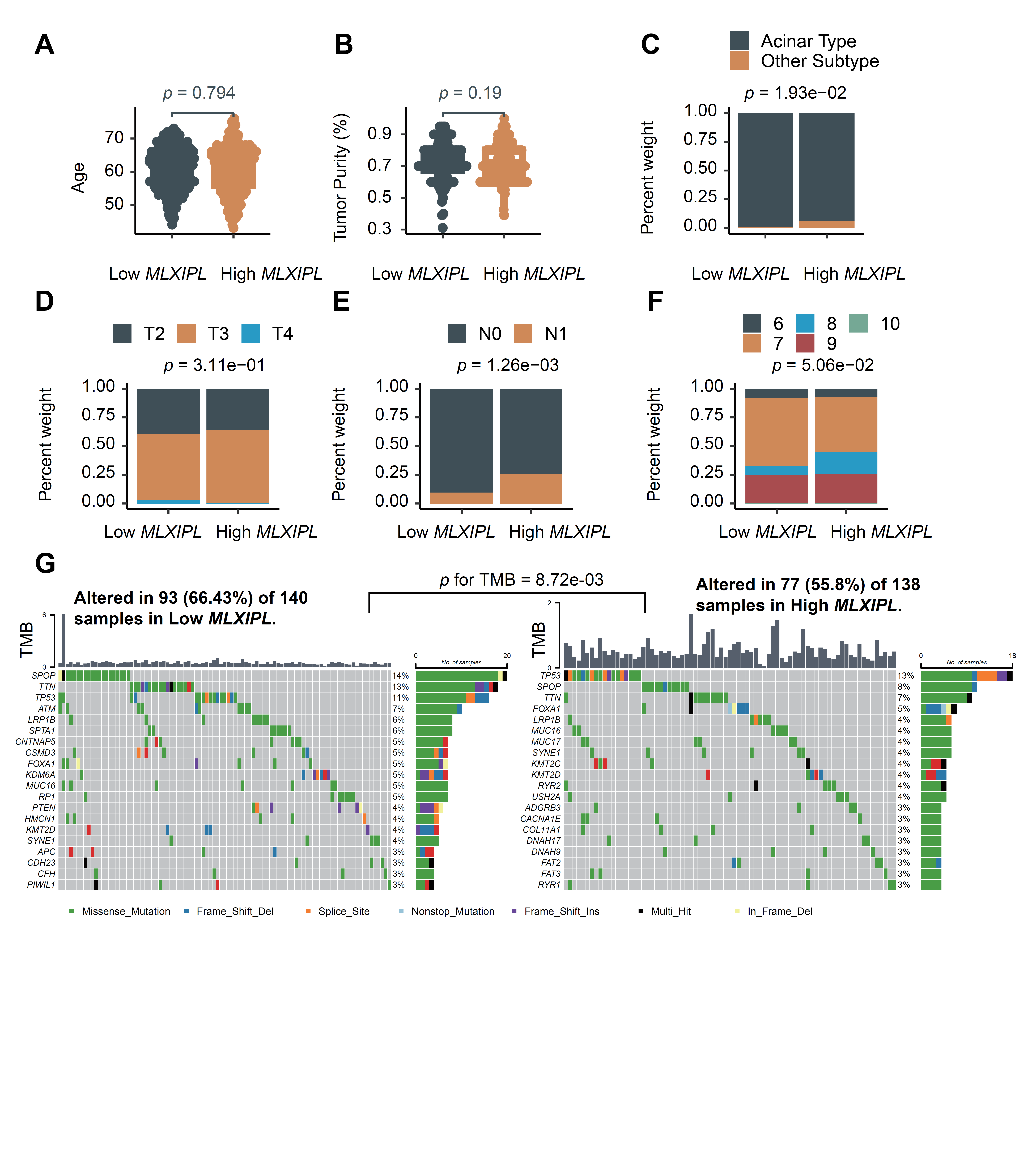


**Supplement Figure 8**


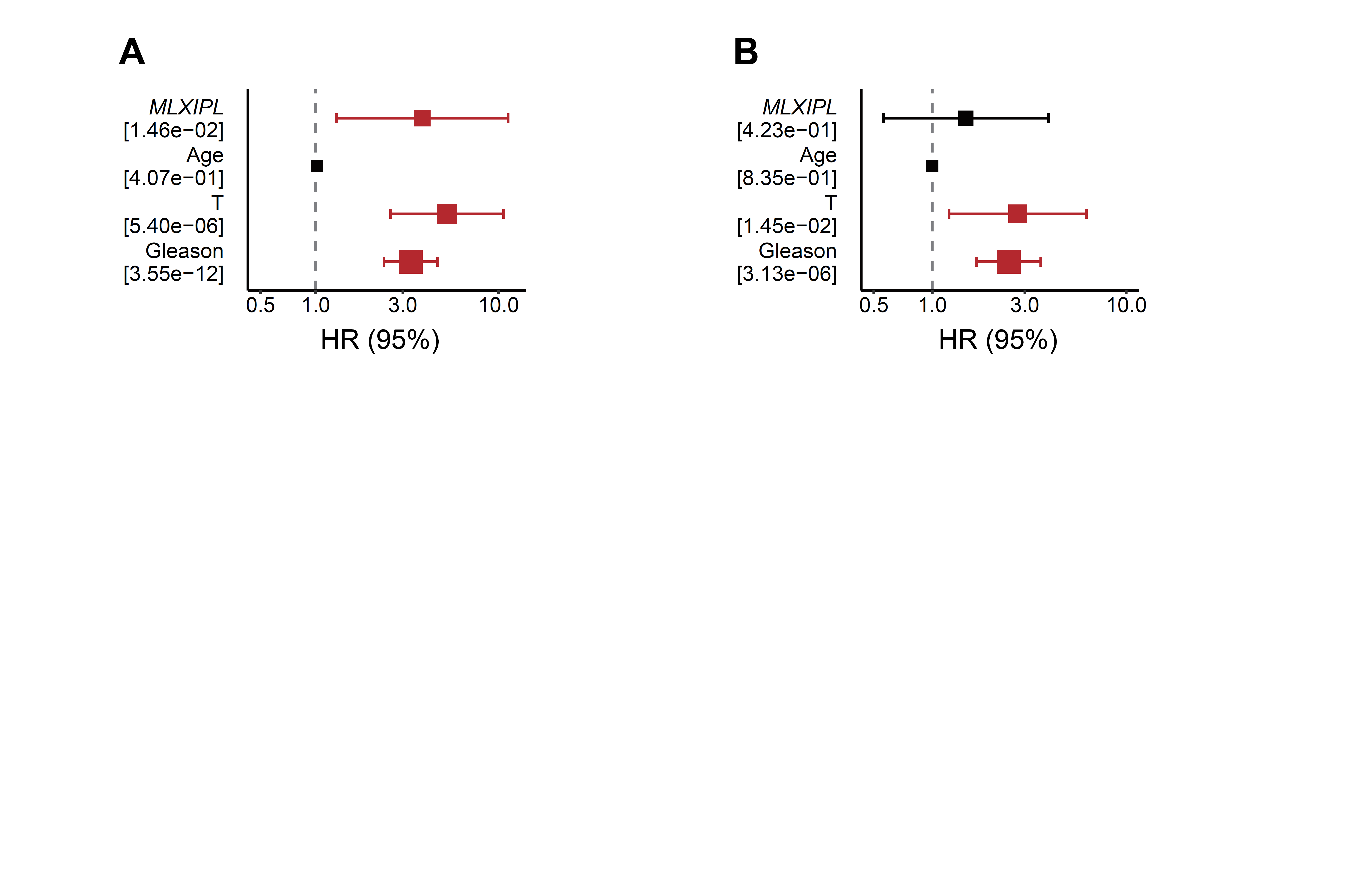


**Supplement Figure 9**


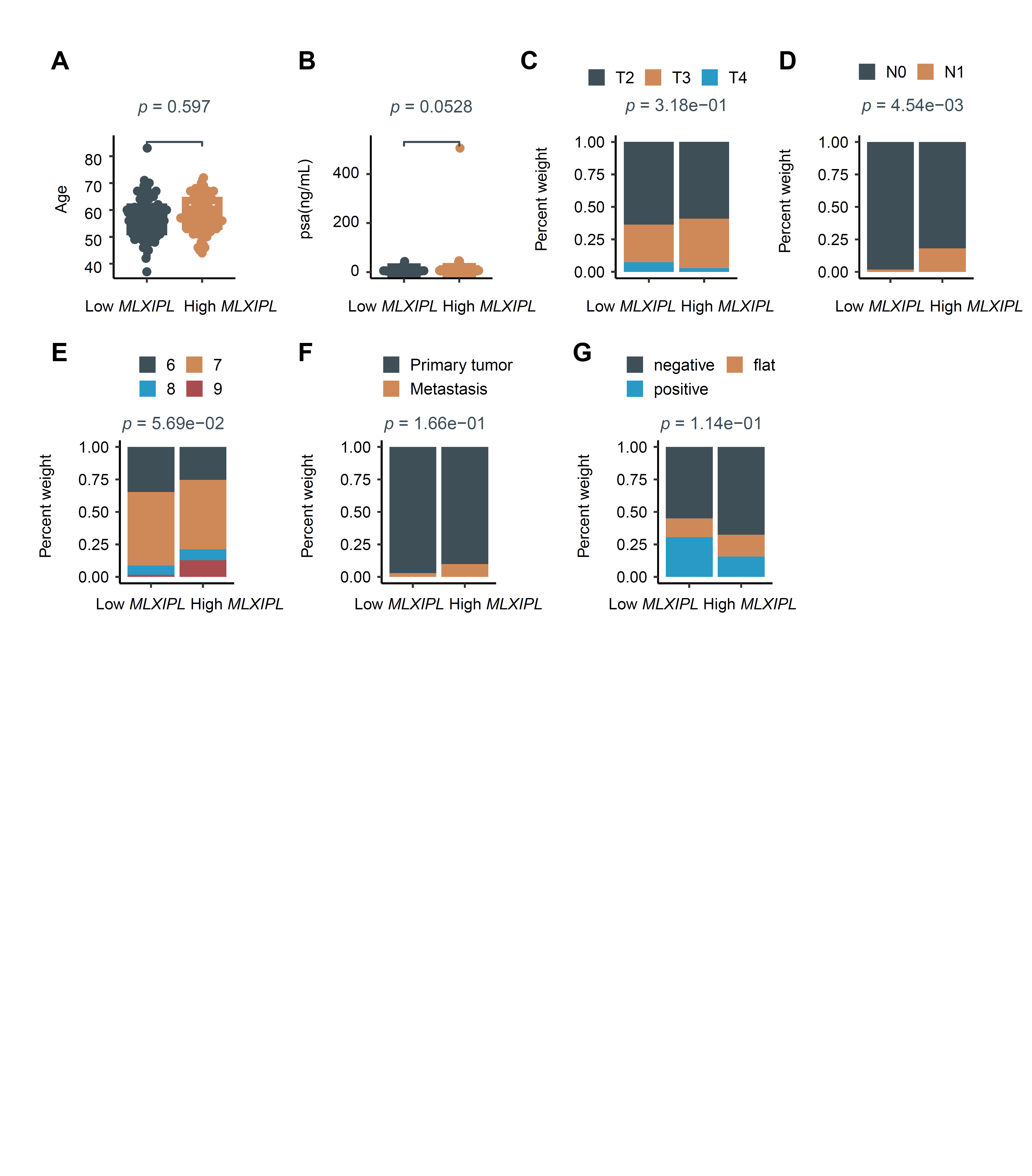


**Supplement Figure 10**


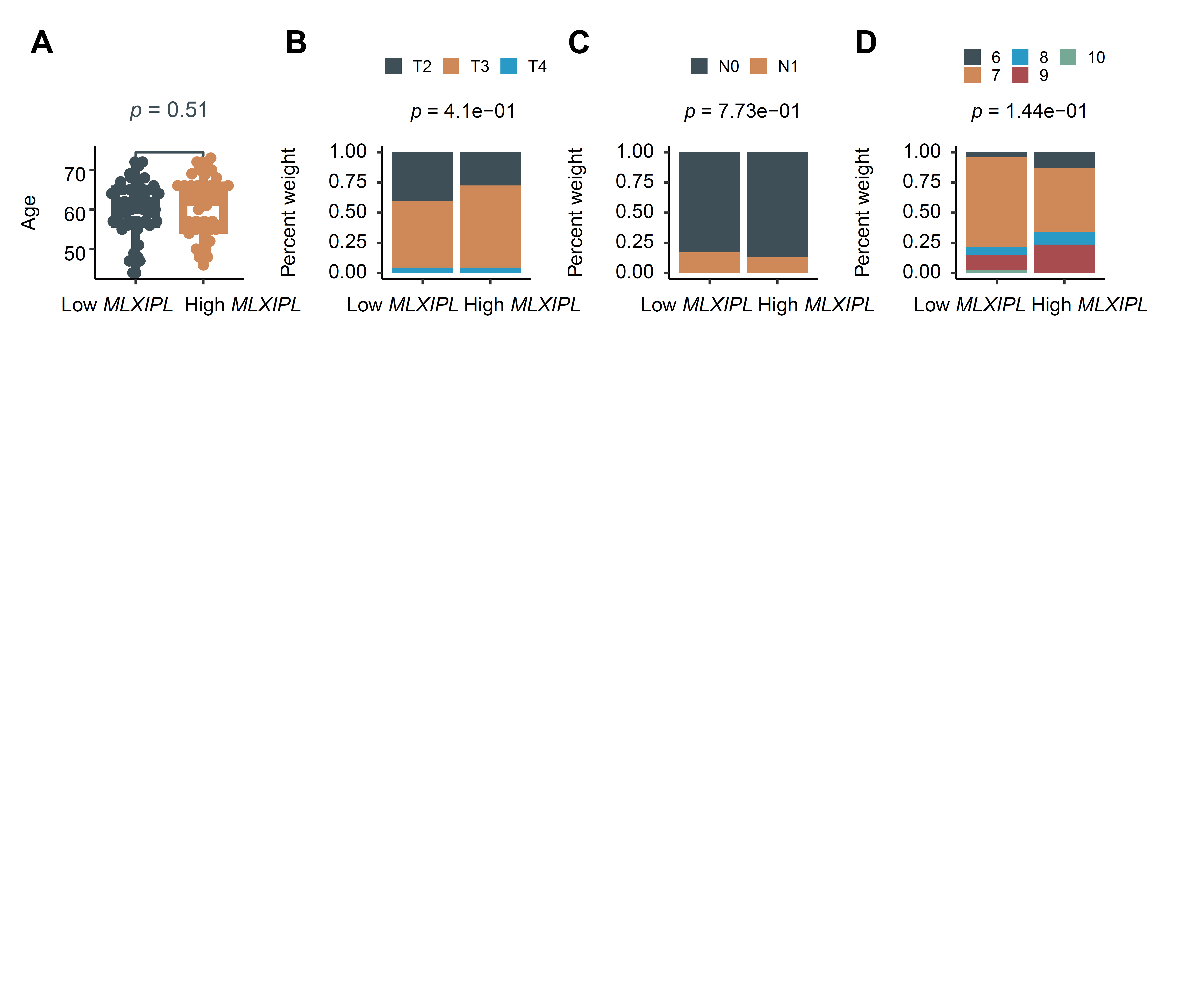


**Supplement Figure 11**


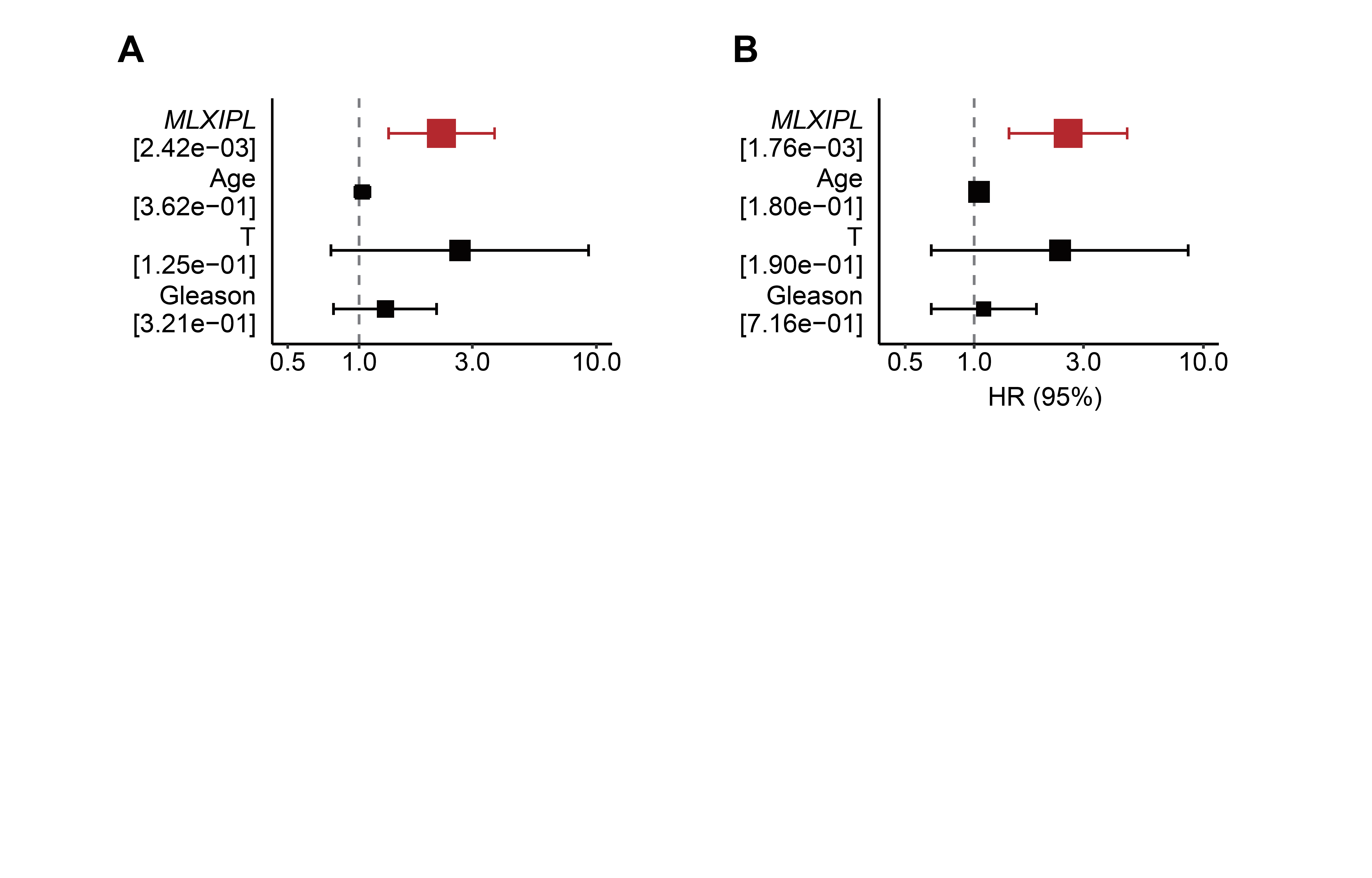


**Supplement Figure 12**


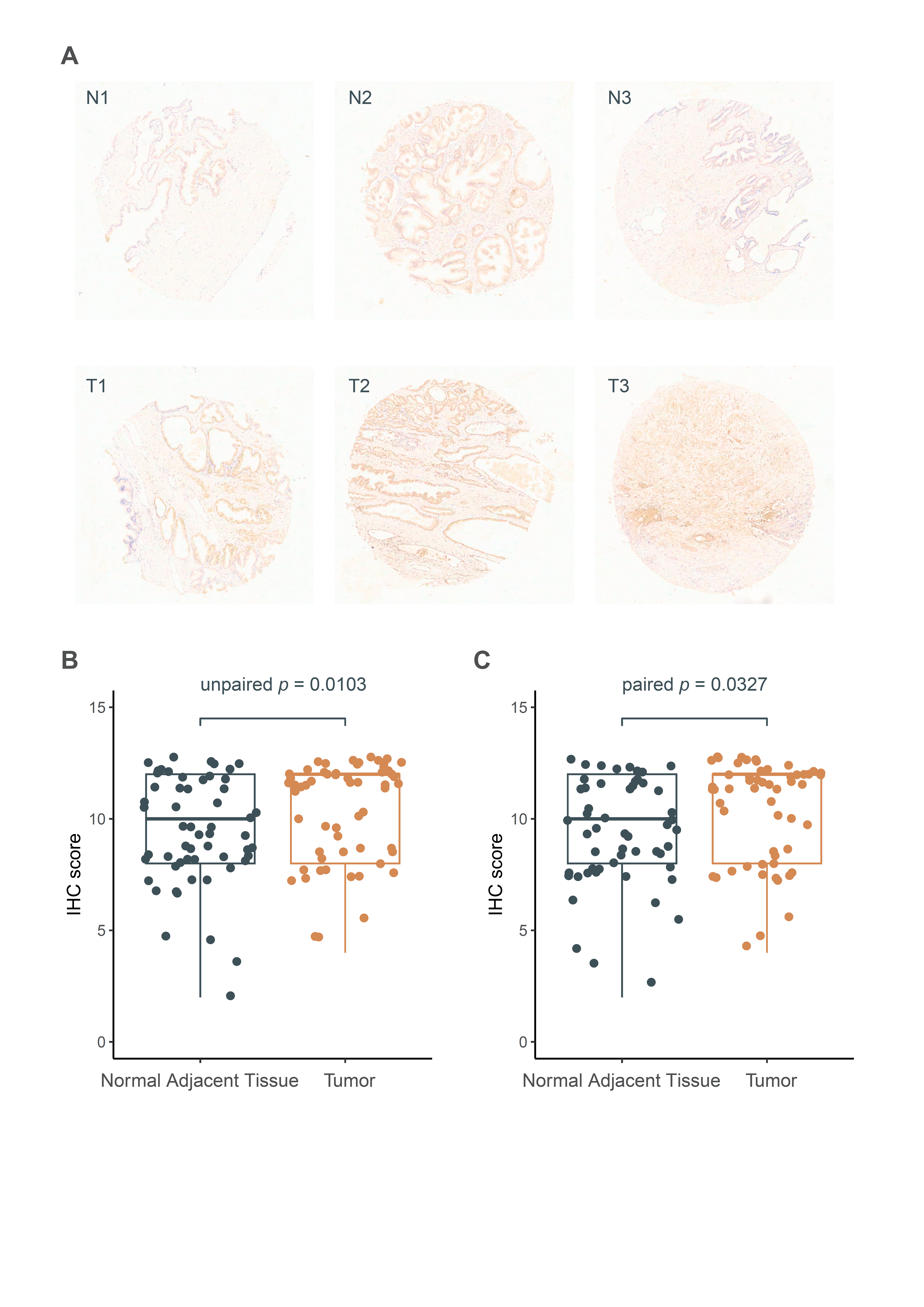

Supplement: Supplementary file 1 [file DataSheet_1.docx]
